# Polyketides with Cardioprotective Bioactivities from Sponge-Associated Fungus *Aspergillus giganteus* MA46-5

**DOI:** 10.3390/molecules30071632

**Published:** 2025-04-06

**Authors:** Ying-Tong Lin, Xiao-Wei Yao, Zheng-Wu Luo, Wei-Xin Jiang, Yin-Fei Wu, Ze-Jun Li, Xue-Wei Duan, Meng-Dan Zhang, Yuan-Yuan Cheng, Cui-Xian Zhang

**Affiliations:** School of Pharmaceutical Sciences, Guangzhou University of Chinese Medicine, Guangzhou 510006, China; lyt2912@163.com (Y.-T.L.); xwyao4834@163.com (X.-W.Y.); 20231110206@stu.gzucm.edu.cn (Z.-W.L.); jiangweixin@simm.ac.cn (W.-X.J.); 20221120250@stu.gzucm.edu.cn (Y.-F.W.); l2941328357@gmail.com (Z.-J.L.); duanxw112@163.com (X.-W.D.); 20231120289@stu.gzucm.edu.cn (M.-D.Z.)

**Keywords:** sponge-associated fungus, *Aspergillus giganteus* MA46-5, polyketides, myocardial protection

## Abstract

One pair of novel enantiomers, gigantdioxin A (+)-**1** and B (−)-**1**, with a skeleton of benzo[*d*][1,3]dioxin; a new acetophenone gigantone A (**3**); a known 3-chlorogentisyl alcohol (**2**), which is the bioprecursor of **1**; acetophenone (**4**); and chromone derivative (**5**) were obtained from the sponge-associated fungus *Aspergillus giganteus* MA46-5. Their structures were established by extensive and in-depth spectral analysis, such as UV, 1D and 2D NMR, and HRESIMS. The absolute configurations of (±)-**1** were deduced by ORD, chiral separation, and experimental and computational ECD. Meanwhile, we proposed a possible biosynthetic pathway of (±)-**1**. Fortunately, the pathway was proved by biomimetic synthesis through **2**, as a bioprecursor, reacted with *n*-butyraldehyde. Myocardial protection assays showed that **3** and **4** possessed stronger protective effects than a positive control against myocardial cell H9c2 ischemia–reperfusion injury in low concentrations, and the effect of (−)-**1** was almost equal to that of the positive control. Further, we explored the possible mechanism of myocardial protection through network pharmacology. Adenosine A2a receptor (ADORA2A) and serum albumin (ALB) represent potential targets for myocardial protection associated with (−)-**1** and **4**, respectively. Based on the network pharmacology, we docked the predicted proteins with bioactive compounds using Autodock Vina.

## 1. Introduction

Examining natural products (NPs) is still an important way to find new therapeutic drugs [1]. The diversity of marine organisms and ecology has led to great potential for the production of natural products [2]. Sponges exhibit a porous structure and acquire nutrients through the ingestion of substantial volumes of water [3]. This predatory behavior facilitates the introduction of microorganisms into their internal canals, rendering them a hotspot for microbial aggregation [4]. The most recent report on marine natural products indicates that *Aspergillus* continued to be a significant source of new metabolites in 2023, reaffirming its status as the primary source of fungal metabolites [5]. *Aspergillus* represents the primary fungal source of novel natural products, while fungi derived from sponges constitute the most prolific source of new natural compounds [6]. Sponge-derived *Aspergillus* strains exhibit exceptional antibacterial properties [7]. Furthermore, *Aspergillus* is an excellent producer of polyketides with therapeutic potential [8]. However, due to the extensive diversity within the class of polyketides, specific research is still needed.

Cardiovascular disease (CVD) is still the leading cause of death, morbidity, and health care expenditure worldwide, and it is one of the most influential diseases in the world [9,10]. Most of the cardiovascular drugs approved by the U.S. FDA from 2011 to 2023 belong to the category of vascular therapy, while the development trend of cardiomyopathy drugs may persist in inadequately addressing medical requirements [11]. Consequently, it is urgent to find new myocardial protective drug leads. The potential of discovering cardiovascular protective drugs from marine microorganisms is underestimated [12].

**Figure 1:**
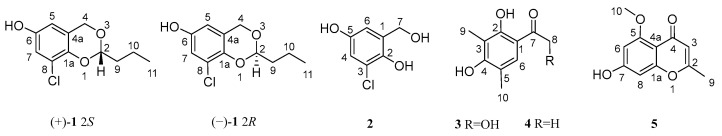
Structures of compounds **1**–**5**.

Previously, our academic group obtained new cardioprotective arylbutenolides: aspergteroids G with disubstituted maleimides from the soft coral-associated symbiotic and epiphytic fungus *Aspergillus terreus* EGF7-0-1 [13]. As part of our ongoing work to explore the active secondary metabolites of marine microorganisms [14,15,16,17], chemical research on sponge-associated *Aspergillus giganteus* MA46-5 was carried out, and ten indolequinazoline alkaloids were isolated from the title strain [18]. In further explorations, three new polyketides were isolated from the title strain MA46-5, of which gigantdioxin **1** is a C-2 enantiomer, and gigantdioxin A (+)-**1** and B (−)-**1** were successfully separated, as well as three known polyketides (Figure 1). We speculated that **2** was a bioprecursor of **1** and confirmed this speculation by biomimetic synthesis. The potential cardioprotective effects of the compounds were evaluated through simulating myocardial ischemia–reperfusion injury at the cellular level and determining possible mechanisms by network pharmacology. Finally, the binding energy, binding site, and interaction of the compounds to the predicted proteins were obtained by molecular docking technology.

## 2. Results

**Figure 2:**
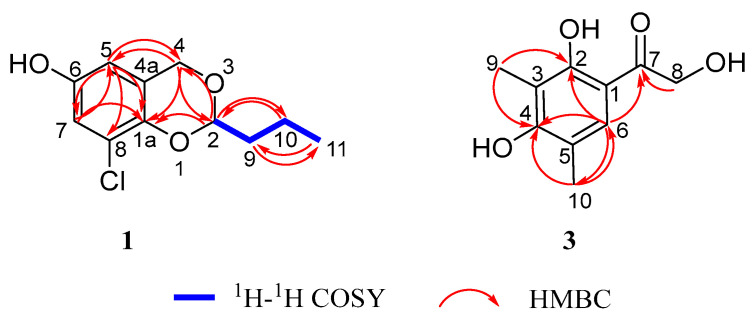
Key HMBC (red arrows) and ^1^H-^1^H COSY (blue bold lines) correlations of compounds **1** and **3**.

**Figure 3:**
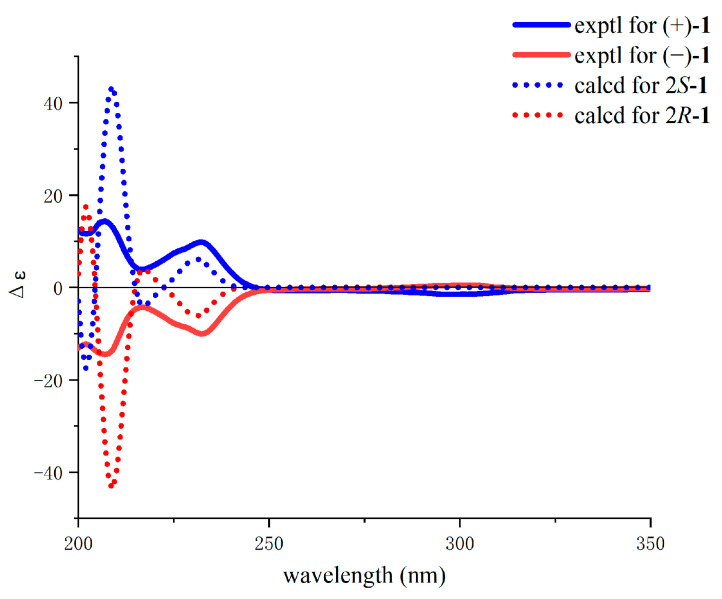
Electronic circular dichroism (ECD) spectra of compounds (+)-**1** and (−)-**1**.

Compound **1** was isolated as a white amorphous powder. Its molecular formula was determined to be C_11_H_13_ClO_3_ by HR-ESIMS ([M-H]^−^ *m*/*z* 227.0486, calcd. for 227.0475, and an isotopic peak at *m*/*z* 229.0461 with a ratio of 3:1), with five degrees of unsaturation. The IR spectrum of **1** showed absorption bands for the hydroxy group at 3363 cm^−1^ and ether group at 1234 and 1220 cm^−1^. The ^1^H NMR spectrum (Table 1) of **1** displayed two aryl hydrogens at *δ*_H_ 6.37 (d, *J* = 2.8, 1H, H-5) and 6.76 (d, *J* = 2.8, 1H, H-7); one dioxymethine hydrogen at *δ*_H_ 4.99 (t, *J* = 5.2, 1H, H-2); one oxymethylene at *δ*_H_ 4.77 (d, *J* = 14.8, 1H, H-4), 4.91 (d, *J* = 14.8, 1H, H-4); and one *n*-propyl at *δ*_H_ 1.86 (m, 2H, H-9), 1.57 (m, 2H, H-10), and 1.00 (t, *J* = 7.4, 3H, H-11). The ^1^H NMR spectrum also showed an exchangeable hydrogen signal at *δ*_H_ 9.40 (brs, 1H, 6-OH). The ^13^C NMR (Table 1) and HSQC spectroscopic data indicated eleven carbon signals comprising two tertiary carbons, *δ*_C_ 110.0 (CH, C-5) and 116.0 (CH, C-7); four quaternary carbons, *δ*_C_ 149.4 (C, C-6), 121.8 (C, C-8), 143.3 (C, C-1a), and 123.1 (C, C-4a); one anomeric carbon, *δ*_C_ 100.6 (CH, C-2); one oxygenated carbon, *δ*_C_ 66.3 (CH_2_, C-4); and three *n*-propyl carbons, *δ*_C_ 36.4 (CH_2_, C-9), 17.2 (CH_2_, C-10), and 14.0 (CH_3_, C-11). ^1^H-^1^H COSY (Figure 2) indicated a spin-coupled system, with correlations of H-2 (*δ*_H_ 4.99, t, *J* = 5.2, 1H) with H-9 (*δ*_H_ 1.86, m, 2H), H-10 (*δ*_H_ 1.57, m, 2H), and H-11 (*δ*_H_ 1.00, t, *J* = 7.4, 3H). It was proved that there was an *n*-butyl structure in the greater structure. HMBC (Figure 2) showed correlations from H-5 (*δ*_H_ 6.37, d, *J* = 2.8, 1H) to C-6 (*δ*_C_ 149.4, C), C-7 (*δ*_C_ 116.0, CH), C-4 (*δ*_C_ 66.3, CH_2_), and C-1a (*δ*_C_ 143.3, C) and from H-7 (*δ*_H_ 6.76, d, *J* = 2.8, 1H) to C-6 (*δ*_C_ 149.4, C), C-8 (*δ*_C_ 121.8, C), C-5 (*δ*_C_ 110.0, CH), and C-1a (*δ*_C_ 143.3, C). From this combined with the effect of different electronegativity of substituents on the chemical shift value, it can be explained that the hydroxyl group and chlorine atom are located at the C-6 and C-8 positions of the aromatic ring, respectively. Furthermore, the HMBC correlation from H-5 (*δ*_H_ 6.37, d, *J* = 2.8, 1H) to C-8 (*δ*_C_ 121.8, C) served as crucial evidence for determining the positions of hydroxyl and chlorine substituents within the molecular structure. Further, the existence of a 1,3-dioxane structure was proved by HMBC correlations from H-4 (*δ*_H_ 4.77, d, *J* = 14.8, 1H) to C-2 (*δ*_C_ 100.6, CH), C-5 (*δ*_C_ 110.0, CH), C-1a (*δ*_C_ 143.3, C), and C-4a (*δ*_C_ 123.1, C); from H-2 (*δ*_H_ 4.99, t, *J* = 5.2, 1H) to C-4 (*δ*_C_ 66.3, CH_2_) and C-10 (*δ*_C_ 17.2, CH_2_); and from H-2 (*δ*_H_ 4.99, t, *J* = 5.2, 1H) to C-1a (*δ*_C_ 143.3, C), which was paralleled with the aromatic ring. Finally, the planar structure of **1** was determined to be gigantdioxin with benzo[*d*][1,3]dioxin. Due to the optical rotation value ${\left[\alpha \right]}_{D}^{25}$ of 0 (*c* 0.20, MeOH), a pair of enantiomeric gigantdioxins, A (+)-**1** and B (−)-**1**, was obtained by chiral separation. As calculated by quantum chemical ECD, the measured values for (+)-**1** and (−)-**1** highly matched with the calculated values for 2*S*-**1** and 2*R*-**1**, respectively. Finally, the absolute configurations of (+)-**1** and (−)-**1** were determined as 2*S*-**1** and 2*R*-**1**, respectively (Figure 3).

**Figure 4:**
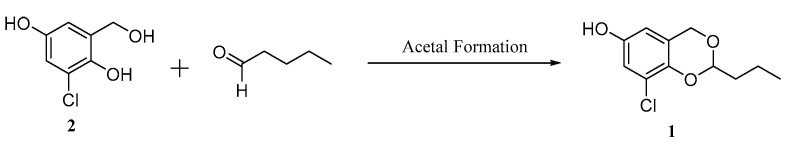
Possible biosynthetic pathway of compounds (±)-**1**.

Furthermore, we speculated as its biosynthetic pathway that it may be obtained from 3-chlorogentisyl alcohol (**2**, also obtained from the same strain) and a molecule of *n*-butyraldehyde through acetal formation (Figure 4). Meanwhile, we found that most of the benzo-1,3-dioxane structures were obtained by chemical synthesis, especially those containing halogens.

Compound **3** was isolated as a colorless needle crystal. Its formula was determined to be C_10_H_12_O_4_ by HR-ESIMS ([M-H]^−^ *m*/*z* 195.0659, calcd. for 195.0657), with five degrees of unsaturation. The IR spectrum of **3** showed absorption bands for the hydroxyl group at 3354 cm^−1^ and carbonyl group at 1624 cm^−1^. The ^1^H NMR (Table 1) spectrum of **3** displayed an aryl hydrogen, *δ*_H_ 7.44 (s, 1H, H-6); six methyl hydrogens, *δ*_H_ 2.01 (s, 3H, H-9) and 2.10 (s, 3H, H-10); two methylene hydrogens, *δ*_H_ 4.71 (s, 2H, H-8); and an exchangeable hydrogen, *δ*_H_ 12.02 (s, 1H, H-2). The ^13^C NMR (Table 1) and HSQC spectroscopic data indicated ten carbon signals comprising five quaternary carbons, *δ*_C_ 109.5 (C, C-1), 160.2 (C, C-2), 110.4 (C, C-3), 160.2 (C, C-4), and 116.2 (C, C-5); a tertiary carbon, *δ*_C_ 128.1 (CH, C-6); a carbonyl carbon, *δ*_C_ 202.7 (C, C-7); two methyl carbons, *δ*_C_ 8.2 (CH_3_, C-9) and 16.3 (CH_3_, C-10); and a methylene carbon, *δ*_C_ 63.9 (CH_2_, C-8). The above NMR information was very similar to that for the acetophenone [19]. Based on the acetophenone, one hydrogen of the C-8 methyl was substituted with a hydroxyl group, and the molecular weight was increased by 16 Da. It was judged that one of the methyl hydrogens was substituted with a hydroxyl group and became methylene. ^1^H-^1^H COSY (Figure 2) showed that compound **3** had no spin-coupled fragments. HMBC (Figure 2) showed correlations from H-6 (*δ*_H_ 7.44, s, 1H) to C-10 (*δ*_C_ 16.3, CH_3_), C-2 (*δ*_C_ 160.2, C), C-4 (*δ*_C_ 160.2, C), and C-7 (*δ*_C_ 202.7, C); from H-9 (*δ*_H_ 2.01, s, 3H,) to C-2 (*δ*_C_ 160.2, C), C-3 (*δ*_C_ 110.4, C) and C-4 (*δ*_C_ 160.2, C,); and from H-10 (*δ*_H_ 2.10, s, 3H) to C-5 (*δ*_C_ 116.2, C), C-6 (*δ*_C_ 128.1, CH), and C-4 (*δ*_C_ 160.2, C). According to the correlation signal of H-8 (*δ*_H_ 4.71, s, 2H) and C-7 (*δ*_C_ 202.7, C), the methylene group was connected to the carbonyl group, and the substitution position of the hydroxyl group was at the C-8 position. Finally, the structure of **3** was determined and named gigantone A.

Three known compounds were also isolated and identified as 3-chlorogentisyl alcohol (**2**), acetophenone (**4**), and chromone derivative (**5**) by comparing their NMR (Table S3) and MS data with previously reported data [19,20,21].

**Scheme 1:**
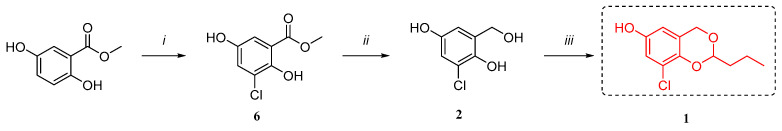
Biomimetic synthesis of (±)-**1**. Reagents and conditions: (**i**) *N*-chlorosuccinimide, DMF, 2.5 h, rt; (**ii**) LiAlH_4_ (2.5 M in THF), THF, 3–5 h, 0 °C → rt → 70 °C; (**iii**) *p*-toluenesulfonic acid, butyraldehyde, THF, 12 h, rt.

In order to verify our speculation (Figure 4), we successfully completed the biomimetic synthesis of **1**, which was also found to be racemic through chiral separation (Scheme 1, Figure S3). 4*H*-Benzo[*d*][1,3]dioxin-4-one and its derivatives are important structural units of insecticides [22]. This synthesis solves the problem of the raw material source to some extent.

**Figure 5:**
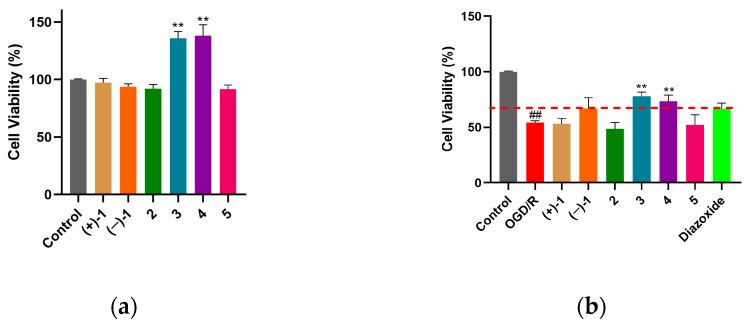
Cell viability assays: (**a**) The toxicity of compounds (±)-**1**–**5** to cardiomyocytes at 10 μM; (**b**) the protective effects of compounds (±)-**1**–**5** on OGD/R-induced cardiomyocytes at 10 μM. Values are expressed as the mean ± SD, *n* = 3. ** *p* < 0.01 vs. control group in (**a**) and vs. OGD/R group in (**b**). ^##^ *p* < 0.01 vs. control group.

Myocardial protection is the focus of attention of the cardiovascular and cerebrovascular system, diseases of which seriously threaten human health and life. Oxygen–glucose deprivation/reperfusion (OGD/R) is a classic model used to simulate myocardial ischemia–reperfusion injury at the cellular level. The results of a normal cardiomyocyte H9c2 toxicity test showed that compounds **1**–**5** had no significant cytotoxicity at 10 μM. Compared with the control group, compounds **3** and **4** significantly increased the viability of H9c2 cells (Figure 5a). Compound **1** showed a tendency to be less toxic to cardiomyocytes than **2**. However, due to the limited number of compounds, the toxicity–structure relationship needs to be further determined. The results of OGD/R-induced cardiomyocyte H9c2 injury protection experiments showed that compounds **3** and **4** had significant protective effects on OGD/R-induced H9c2 injury at 10 μM that were better than those of the positive control (diazoxide). The myocardial protective activity of (−)-**1** was comparable to that of diazoxide (Figure 5b, red dotted line). However, (+)-**1**, **2**, and **5** had no significant cardioprotective activity (Figure 5b). This indicates that the stereo configuration on C-2 of **1** may have an effect on cardioprotective activity. The cardioprotective activity of **3** showed a tendency to be better than that of **4**, which may be related to the introduction of a hydroxyl group at the terminal hydroxymethyl. Based on the insecticidal activity of the benzo[*d*][1,3]dioxin scaffold in agriculture [22], we could expand its potential cardioprotective effect in biomedicine.

**Figure 6:**
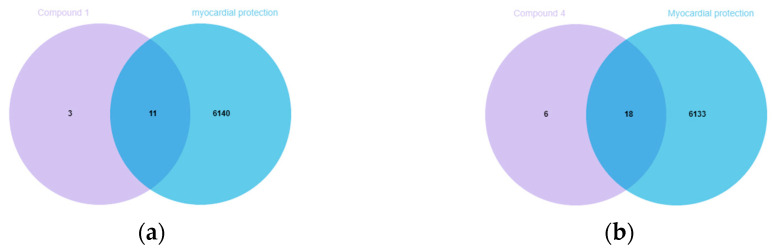
Venn diagrams: (**a**) potential targets of (−)-**1** intersected with myocardial protection-related targets; (**b**) potential targets of **4** intersected with myocardial protection-related targets.

In order to investigate the possible mechanism of the compounds with cardioprotective activities, the potential targets of these compounds were obtained by Swiss Target Prediction [23], but it failed to find corresponding targets for **3**. In the Venn diagrams [24], there were 11 overlapping targets between myocardial protection-related targets and (−)-**1**, as well as 18 overlapping targets with **4** (Figure 6).

**Figure 7:**
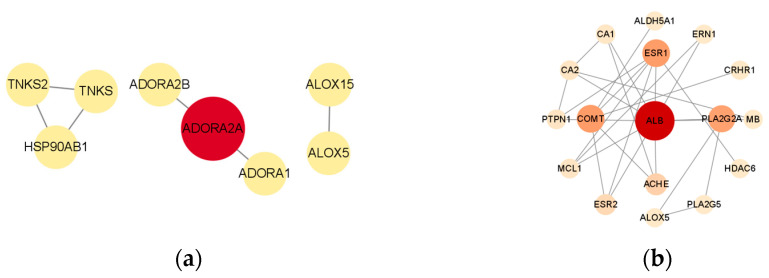
PPI network: (**a**) (−)-**1** and myocardial protection; (**b**) **4** and myocardial protection. The darker the node color, the higher the corresponding protein score.

The protein–protein interaction (PPI) network diagram for the aforementioned cross-targets was sourced from the STRING database [25]. The PPI network diagram was subsequently processed and analyzed using Cytoscape 3.9.1 [26]. The results indicated that adenosine A2a receptor (ADORA2A) may serve as a potential cardioprotective target for (−)-**1** (Figure 7a), while serum albumin (ALB) may represent a potential cardioprotective target for **4** (Figure 7b). The activation of ADORA2A promotes cell survival by inhibiting apoptosis and autophagy, thereby mitigating myocardial ischemia–reperfusion injury (MIRI), which may be associated with its capacity to activate the cAMP-PKA pathway and the Beclin-1-Bcl-2 complex [27]. ALB, COMT, and ESR1 represent significant myocardial targets, and there have been some studies on their mechanisms [28,29]. We offer a fundamental source and thoughts concerning the ligands and proteins pertinent to research on myocardial protection.

After that, a molecular docking method was used to predict the binding affinity of ligands and receptor proteins. ADORA2A was used as the receptor protein of (−)-**1**. The ADORA2A agonist regadenoson [30] was used as a positive control. Compound **4** docked with the first three high-score proteins, ALB, COMT, and ESR1. Since the structures of **3** and **4** are similar, we also tried to dock **3** with ALB, COMT, and ESR1. Autodock Vina was used to obtain the binding energies of the compounds and proteins (Table 2). The results showed that the binding energies of **3** to the proteins were close to or even lower than those of **4**. The binding energies of (−)-**1** to the corresponding proteins were higher than those of **4**, which seems to be consistent with the results of the cell assay in which the myocardial protective activity of **4** was stronger than that of (−)-**1**. This indicates that the predicted targets may be responsible for the results of the myocardial cell test.

**Figure 8:**
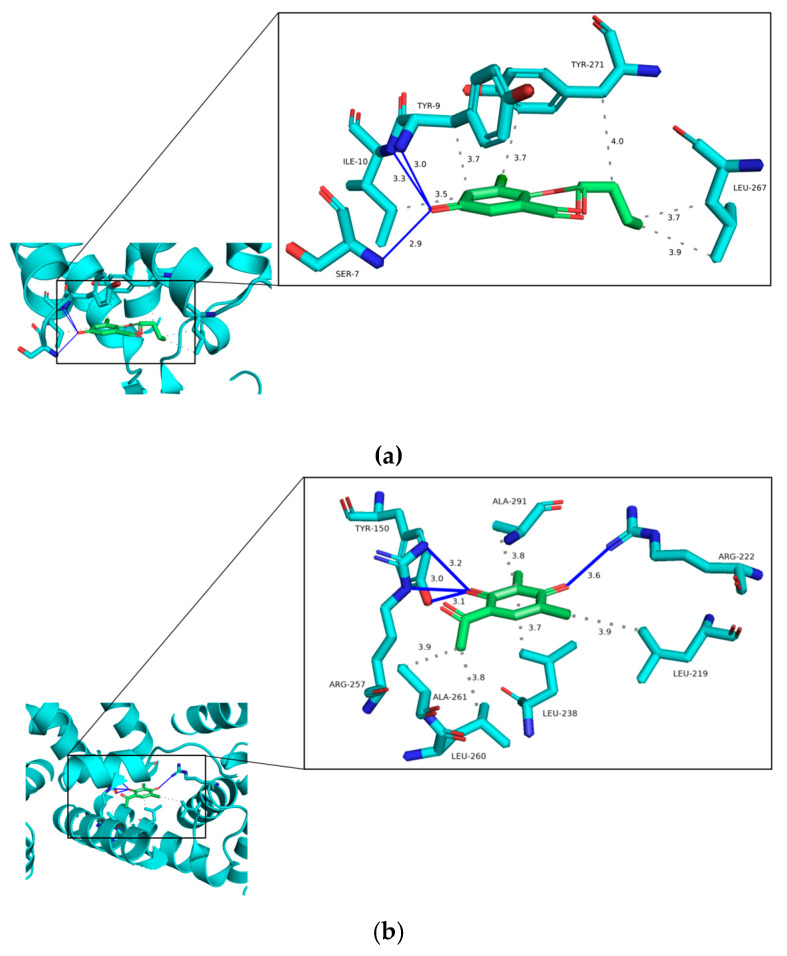
The binding interactions of ligands with proteins: (**a**) (−)-**1** with ADORA2A (PDB:3PWH); (**b**) **4** with ALB (PDB:1AO6). Blue solid line: hydrogen bond; gray dotted line: hydrophobic interaction. Ligands are represented as green stick models, proteins are presented as cartoon models.

Based on the docking results, we further discuss the binding interactions of (−)-**1** and **4** with the proteins with the lowest binding energy (Figure 8). The binding energy between (−)-**1** and ADORA2A (PDB:3PWH) is −6.00 kcal·mol^−1^. The results of the interaction showed that the ligand (−)-**1** binds to the residues TYR-9, ILE-10, and SER-7 by hydrogen bonds with distances ranging from 2.9 Å to 3.3 Å. In addition, (−)-**1** has hydrophobic interactions with TRY-9, ILE-10, LEU-267, and TRY-271 with distances ranging from 3.5 Å to 4.0 Å (Figure 8a). The binding energy of **4** to ALB (PDB: 1AO6) is −6.80 kcal·mol^−1^. As can be seen (Figure 8b), **4** binds to the residues TYR-150, ARG-257, and ARG-222 by hydrogen bonds with distances ranging from 3.0 Å to 3.6 Å. Meanwhile, **4** has hydrophobic interactions with LEU-219, LEU-238, LEU-260, ALA-261, and ALA-291 with distances ranging from 3.7 Å to 3.9 Å.

## 3. Materials and Methods

### 3.1. General Experimental Procedures

Optical rotation was measured on an MCP 200 digital polarimeter (Anton Paar, Graz, Austria). UV and circular dichroism were determined on a Circular Dichroism J-810 (JASCO, Tokyo, Japan). The infrared spectra were tested on an IR Affinity-1 infrared spectrometer and an IRTracer-100 Fourier transform infrared spectrophotometer (Shimadzu Corporation, Kyoto, Japan). ^1^H NMR, ^13^C NMR, and two-dimensional NMR spectra were recorded on a Bruker AVANCE III HD 400 MHz DIGITAL NMR spectrometer (Bruker Optics, Karlsruhe, Germany) with TMS as the internal standard. HR-ESI-MS and MS/MS data were obtained by using a Triple TOF^TM^ 5600+ system (AB SCIEX, Framingham, MA, USA). Column chromatography (CC) was performed with 200–300-mesh silica gel (Qingdao Marine Chemical Factory, Qingdao, China) and Sephadex LH-20 (Cytiva, Uppsala, Sweden). TLC was performed on GF254 silica gel (Qingdao Marine Chemical Factory, Qingdao, China), and spots were visualized under an ultraviolet transmission reflectometer (Shanghai Jingke Industrial, Shanghai, China). Preparative HPLC was performed on a Semipreparative HPLC device (QuikSep, Beijing, China) with a Kromasil 100–10 C18 column (250 × 21.2 mm, 10 μm, Bohus, Sweden), Chiral ND (2) NFC 5u (250 × 10 mm, 5 μm, Guangzhou, China), and H&E sp ODS-A (250 × 10 mm, 5 μm, Beijing, China). The reagents related to the synthesis were purchased from commercial suppliers (Aladdin, Shanghai Aladdin Biochemical Technology Co., Ltd., Shanghai, China; Macklin, Shanghai Macklin Biochemical Co., Ltd., Shanghai, China; Energy, Anhui Zesheng Technology Co., Ltd., Anhui, China) and used directly without further purification.

### 3.2. Strain Material

The fungus *Aspergillus giganteus* MA46-5 was isolated from a marine sponge collected from the Beibu Gulf of China (Weizhou Island, Beihai City, Guangxi Zhuang Autonomous Region). The DNA of the strain was extracted, and the ribosomal ITS gene region of the fungus was amplified by PCR. The results were compared with the known sequences in the GenBank database on the NCBI website. The strain was identified as *Aspergillus giganteus* (sequence number MT529399, homology 100%). The strain (No. MA46-5) is preserved in the Marine Natural Medicine Laboratory, School of Pharmaceutical Sciences, Guangzhou University of Chinese Medicine.

### 3.3. Fermentation, Extraction, and Isolation

The strain *A. giganteus* MA46-5 was cultured in a 1000 mL culture flask containing solid rice medium and PDB medium at room temperature under static conditions for 35 days. The composition of the PDB medium consisted of 200 g of potato per liter, 20 g of glucose per liter, and 3.3% sea salt. A total of 400 mL of PDB medium was placed into each 1 L culture bottle, resulting in 1000 bottles overall. The solid rice culture medium was made up of 100 g of rice per 1 L culture bottle, along with 3.3% sea salt and 115 mL of distilled water per bottle, resulting in a total of 500 bottles.

The solid rice medium was successively extracted with ethyl acetate (300 mL/bottle/time, twice) and methanol (300 mL/bottle/time, once). The ethyl acetate extract was combined and concentrated under reduced pressure to obtain EtOAc-1 extract (540 g). The methanol extract was concentrated under reduced pressure and dispersed in water, then extracted with ethyl acetate and *n*-butanol in turn. The ethyl acetate extract was concentrated under reduced pressure to obtain EtOAc-2 extract (15 g). The results of TLC/LC-MS and NMR analyses showed that EtOAc-1 and -2 could be combined to obtain EtOAc extract (555 g). The PDB medium was filtered through gauze to obtain the fungal supernatant and mycelium, and the fungal supernatant was concentrated by the water bath heating method. The fungal supernatant was extracted three times with ethyl acetate and *n-*butanol in turn (*v*:*v* = 1:1), and the combined solution was concentrated under reduced pressure to obtain *n*-BuOH-1 (247 g). The mycelia were extracted with methanol seven times at room temperature and finally extracted once by the heating reflux method. The methanol extract was concentrated under reduced pressure, and then the extract was dispersed in water. The extract was extracted three times with ethyl acetate and *n*-butanol (*v*:*v* = 1:1), and the combined solution was concentrated under reduced pressure to obtain *n*-BuOH-2 (36 g). The results of TLC and LC-MS showed that *n*-BuOH-1 and -2 could be combined to obtain *n*-BuOH extract (283 g).

The ethyl acetate extract from the solid rice medium was separated by silica gel column chromatography, gradiently eluted with 100:00–00:100 (*v*/*v*) petroleum ether/ethyl acetate solvent, and finally eluted with methanol. The separation process was monitored by TLC, and the spot position was observed under an ultraviolet lamp (254 nm and 365 nm). Subsequently, it was combined into 12 sub-fractions (Fr.1–Fr.12). Fr.3 (8 g) was separated by silica gel column chromatography with gradient elution using 100:00–00:100 (*v*/*v*) of petroleum ether/ethyl acetate solvent and then washed with methanol. The separation process was monitored by TLC, and the spot position was observed under an ultraviolet lamp (254 nm and 365 nm). Subsequently, it was combined into 7 fractions (Fr.3.1–Fr.3.7). Fr.3.3 (3.2 g) was fractionated by Sephadex LH-20 column chromatography to obtain seven sub-fractions (Fr.3.3.1–Fr.3.3.7). Fr.3.3.7 (80 mg) was purified by HPLC [H&E C18 (10 × 250 mm, 5 μm, Beijing, China), MeOH/H_2_O 48%, *v*/*v*, 2 mL/min] to yield **3** (*t*_R_ = 27 min, 11 mg). In addition, the Fr.2 (37.5 g) precipitate solid was **4** (18.5 g) and the Fr.5 (23 g) precipitate solid was **5** (4 mg). The methanol-soluble fraction (200 g) of the *n*-BuOH extract in PDB medium was fractionated by silica gel column chromatography and gradiently eluted with 100:00–00:100 (*v/v*) dichloromethane/methanol solvent. The separation process was monitored by TLC, and the spot position was observed under an ultraviolet lamp (254 nm and 365 nm). Subsequently, it was combined into 9 sub-fractions (Fr.1–Fr.9). The Fr.5 (18.4 g) precipitate solid was **2** (30 mg). Fr.3 (500 mg) was purified by HPLC [Kromasil 100-10 C18 column (250 × 21.2 mm, 10 μm, Bohus, Sweden), MeOH/H_2_O 72%, *v/v*, 5 mL/min] to yield **1** (*t*_R_ = 39 min, 5 mg). The enantiomeric separation of **1** by HPLC [Chiral ND (2) NFC 5u (250 × 10 mm, 5 μm, Guangzhou, China), *n*-Hexane/isopropanol 70/30, *v/v*, 2 mL/min] yielded (+)-**1** (*t*_R_ =10.1 min, 2.3 mg) and (−)-**1** (*t*_R_ = 16.1 min, 2.1 mg) (for the HPLC spectrum, see Figure S2).

### 3.4. Cell Viability Assays

H9c2 was derived from ATCC (Guangzhou Ubigene Biotechnology Co., Ltd., Guangzhou, China). The cells were cultured in complete high-glucose DMEM (containing 10% FBS, 1% PS) in a 5% CO_2_, 37 °C, saturated-humidity incubator. The medium was changed once every two days; the cells were passaged for 3~5 days and digested with EDTA trypsin.

H9c2 cells in logarithmic phase and in a good growth condition were digested with trypsin and inoculated into 96-well plates at a density of 8 × 104 cells per well. After cell attachment, the original medium was discarded. In the cytotoxicity experiment, 100 μL of drug-containing (10 μM test compounds) complete medium was added to each well, and the cells were cultured for 24 h. The cell viability assay was performed with OGD/R treatment: 100 μL of sugar-free medium was added, and the cells were placed in an anoxic device for 6 h of oxygen–glucose deprivation. The medium was replaced with 100 μL of drug-containing (10 μM test compounds and 100 μM diazoxide) complete medium, and the cells were cultured in a normal incubator for 12 h. The MTT assay was used to test the cell viability: the original medium was discarded, the prepared MTT working solution (prepared with complete medium, concentration of 0.5 mg·mL^−1^) was added at 100 μL per well, and the cells were incubated at 37 °C for 4 h. After that, the MTT working solution was removed, DMSO was added at 150 μL per well, and the mixture was shaken on an oscillator for 10 min in the dark.

### 3.5. Network Pharmacology

The compound targets were sourced from Swiss Target Prediction (http://www.swisstargetprediction.ch/ (accessed on 6 December 2024)). By utilizing the search term “myocardial protection”, a total of 6073 and 108 targets were identified in the GeneCards (https://www.genecards.org/ (accessed on 9 October 2024)) and OMIM (https://www.omim.org/ (accessed on 9 October 2024)), respectively. Following deduplication, a total of 6151 targets related to myocardial protection were compiled. The identification of compounds and disease cross-targets was facilitated through the use of a Venn diagram. The protein–protein interaction (PPI) network was constructed using the STRING database (https://cn.string-db.org/ (accessed on 16 December 2024)). Subsequently, the PPI networks were processed using Cytoscape 3.9.1, and the CytoNCA plugin was employed to analyze the results by ranking the nodes based on their Betweenness Centrality.

### 3.6. Molecular Docking

Based on network pharmacology, proteins were sourced from the Protein Data Bank (PDB) (https://www.rcsb.org/). Ligand optimization and format conversion were performed using Chem 3D 22.0.0.22. AutoDockTools-1.5.6 was utilized for dehydration, hydrogenation, ligand root determination, the selection of ligand twistable bonds, and molecular docking. After preprocessing, the ligand and protein were loaded into AutoDockTools-1.5.6 to perform docking by running Autodock Vina. Autodock Vina subordinates to AutoDockTools-1.5.6. The Protein–Ligand Interaction Profiler (PLIP) [31] provided the interaction network. pyMOL 2.2.0 was used to analyze the docking results.

### 3.7. Structural Characterizations of 1 and 3

Gigantdioxin (±)-**1**: White amorphous powder, UV (MeOH) *λ*_max_ 200 and 300 nm. IR: 3363 cm^−1^ (O-H), 1653 cm^−1^ (phenyl ring), 1589 cm^−1^ (phenyl ring), 1487 cm^−1^ (phenyl ring), 1465 cm^−1^ (phenyl ring), 1234 cm^−1^ (C-O-C), and 1220 cm^−1^ (C-O-C). ^1^H NMR (CDCl_3_, DMSO-*d*_6_, 400 MHz) and ^13^C NMR (CDCl_3_, 100 MHz): given in Table 1; HRESIMS: *m*/*z* [M-H]^−^ 227.0486 (calcd. for C_11_H_12_ClO_3_^−^: 227.0475), with an error margin of 4.8 ppm.

Gigantdioxin A (+)-**1**: ${\left[\alpha \right]}_{D}^{25}$ + 67.3 (*c* 0.10, MeOH); ECD (MeOH) *λ*_max_ (Δε) 207 (14.4), 232 (9.8), 299 (−1.5) nm.

Gigantdioxin B (−)-**1**: ${\left[\alpha \right]}_{D}^{25}$ − 69.4 (*c* 0.10, MeOH); ECD (MeOH) *λ*_max_ (Δε) 207 (−14.5), 232 (−10.0), 302 (0.5) nm.

Gigantone A: Colorless needle crystal. IR: 3354 cm^−1^ (O-H), 1624 cm^−1^ (C=O), 1489 (phenyl ring), 1506 (phenyl ring), and 1541 cm^−1^ (phenyl ring). ^1^H NMR (DMSO-*d*_6_, CDCl_3_, 400 MHz) and ^13^C NMR (DMSO-*d*_6_, 100 MHz): given in Table 1; HRESIMS: *m*/*z* [M-H]^−^ 195.0659 (calcd. for C_10_H_11_O_4_^−^: 195.0657), with an error margin of 1.0 ppm.

### 3.8. General Procedure for Synthesis

#### 3.8.1. The Synthesis of Methyl 3-chloro-2,5-dihydroxybenzoate (6, Scheme 1)

*N*-chlorosuccinimide (794.1 mg, 5.95 mmol, 1.0 eq) was slowly added to a solution of methyl 2,5-dihydroxybenzoate (1.0 g, 5.95 mmol, 1.0 eq) in DMF (10 mL). After the reaction mixture was stirred for 2.5 h at rt under a N_2_ atmosphere, it was quenched with water (10 mL). The mixture was extracted with acetate (3 × 10 mL), and the combined organic layers were washed with water (3 × 10 mL), dried over sodium sulfate, and concentrated *in vacuo*. Purification of the crude product by silica gel column chromatography (100% CH_2_Cl_2_) afforded **6** (800.0 mg, 3.95 mmol) as a pale yellow solid. Yield: 66.7%. ^1^H NMR (400 MHz, CDCl_3_) *δ* 10.85 (s, 1H), 7.24 (s, 1H), 7.15 (s, 1H), 4.71 (s, 1H), 3.96 (s, 3H). ^13^C NMR (100 MHz, CDCl_3_) *δ* 170.0(C), 151.9(C), 147.4(C), 124.3(CH), 122.7(C), 114.1(CH), 113.3(C), 53.0(CH_3_).

#### 3.8.2. The Synthesis of 3-chlorogentisyl Alcohol (2, Scheme 1)

LiAlH_4_ (1.18 mL, 3.00 mmol, 6.0 eq, 2.5 M in THF) was slowly added to a solution of **6** (100.0 mg, 0.50 mmol, 1.0 eq) in THF (3mL) at 0 °C under a N_2_ atmosphere. After the addition was complete, the reaction mixture was stirred at room temperature for 10 min and then refluxed at 70 °C for 3–5 h. After the end of the reaction was monitored with phosphomolybdic acid staining and the mixture was cooled to 0 °C, a sat. aq. solution of NH_4_Cl was slowly added until no gas was produced. The reaction mixture was extracted with ethyl acetate (10 mL × 3), dried over sodium sulfate, and concentrated *in vacuo*.

After extraction, the residue was dispersed with 5 mL of methanol, filtered to remove the solid, then washed with methanol (5 mL) and concentrated *in vacuo*. Purification of the crude product by silica gel column chromatography (CH_2_Cl_2_:CH_3_OH = 100:1) afforded **2** (50.0 mg, 0.29 mmol) as a white solid. Yield: 58%. ^1^H NMR (400 MHz, methanol-*d*_4_) *δ* 6.73 (d, *J* = 2.9 Hz, 1H), 6.66 (d, *J* = 2.9 Hz, 1H), 4.62 (s, 2H). ^13^C NMR (100 MHz, methanol-*d_4_*) *δ* 151.8(C), 144.3(C), 132.1(C), 121.9(C), 115.4(CH), 114.5(CH), 61.1(CH_2_).

#### 3.8.3. The Synthesis of benzo[d][1,3]dioxin Gigantdioxin (1, Scheme 1)

Butyraldehyde (10 µL, 0.11 mmol, 1.2 eq) and *p*-toluenesulfonic acid (3.0 mg, 0.02 mmol, 0.2 eq) were added to a solution of **2** (15.0 mg, 0.09 mmol, 1.0 eq) in THF (2 mL). The reaction mixture was stirred for 12 h at rt under a N_2_ atmosphere. The mixture was then subjected directly to silica gel column chromatography (CH_2_Cl_2_, 100%), affording **1** (5.0 mg, 0.02 mmol) as a white solid. Yield: 25.4%. ^1^H NMR (400 MHz, CDCl_3_) *δ* 6.76 (d, *J* = 2.8 Hz, 1H), 6.37 (d, *J* = 2.8 Hz, 1H), 4.99 (t, *J* = 5.2 Hz, 1H), 4.91 (d, *J* = 14.8 Hz, 1H), 4.77 (d, *J* = 14.8 Hz, 1H), 1.86 (m, 2H), 1.57 (m, 2H), 1.00 (t, *J* = 7.4 Hz, 3H). ^13^C NMR (100 MHz, CDCl_3_) *δ* 149.3(C), 143.4(C), 123.1(C), 121.8(C), 116.0(CH), 110.0(CH), 100.6(CH), 66.3(CH_2_), 36.4(CH_2_), 17.2(CH_2_), 14.0(CH_3_).

## 4. Conclusions

In summary, three novel polyketides and three known polyketides were isolated from the sponge-associated fungus *Aspergillus giganteus* MA46-5. Among them, (±)-**1** is an enantiomer at the C-2 position, and it was speculated that it may be obtained from **2** and a molecule of *n*-butyraldehyde through acetal formation to remove a molecule of H_2_O. After that, we proved this inference by biomimetic synthesis. Furthermore, (−)-**1**, **3**, and **4** showed cardioprotective activities. The results of network pharmacology showed that adenosine A2a receptor is a possible cardioprotective target of (−)-**1**, and serum albumin is a possible cardioprotective target of **4**. Utilizing molecular docking technology, the binding energy, binding site, and interaction were predicted in silico test. The results of the silico test indicated that the binding energy of (−)-**1** to its predicted optimal target is higher than that of **4** to its corresponding optimal target, which seems to explain the result of the cardioprotective effect of **4** being stronger than that of (−)-**1** in the first cell assay.

This study provides novel compound scaffold information and potential target information sources for the discovery of cardioprotective lead compounds, which may support further chemical and core mechanistic research on myocardial protection to some extent.

## Figures and Tables

**Table 1:** ^1^H and ^13^C NMR spectral data of (±)-**1** in CDCl_3_ and **3** in DMSO-*d*_6_ (*δ* in ppm, *J* in Hz).

No.	1		3	
	*δ*_H_, Mult, *J*	*δ*_C_, Type	*δ*_H_, Mult, *J*	*δ*_C_, Type
1				109.5, C
1a		143.3, C		
2	4.99, t, (5.2)	100.6, CH		160.2, C
3				110.4, C
4	4.91, d, (14.8)	66.3, CH_2_		160.2, C
	4.77, d, (14.8)			
4a		123.1, C		
5	6.37, d, (2.8)	110.0, CH		116.2, C
6		149.4, C	7.44, s	128.1, CH
7	6.76, d, (2.8)	116.0, CH		202.7, C
8		121.8, C	4.71, s	63.9, CH_2_
9	1.86, m	36.4, CH_2_	2.01, s	8.2, CH_3_
10	1.57, m	17.2, CH_2_	2.10, s	16.3, CH_3_
11	1.00, t, (7.4)	14.0, CH_3_		
2-OH			^b^ 12.02, s	
4-OH			n.o.	
6-OH	^a^ 9.40, s			
8-OH			n.o.	

^a^ The solvent was replaced with DMSO-*d*_6_ to measure the signal. ^b^ The solvent was replaced with CDCl_3_ to measure the signal. “n.o.” means “not observed”.

**Table 2:** Binding energies of compounds to proteins.

Compounds	Ebind (kcal·mol^−1^)				
	ADORA2A (PDB:2YDO)	ADORA2A (PDB:3PWH)	ALB (PDB:1AO6)	COMT (PDB:4PYI)	ESR1 (PDB:6VPF)
(−)-**1**	−5.80	−6.00	-	-	-
Regadenoson	−7.60	−7.10	-	-	-
**3**	-	-	−6.80	−6.80	−6.70
**4**	-	-	−7.00	−6.60	−6.10

## Data Availability

Data are contained within the article and Supplementary Materials.

## Supplementary Materials

The following supporting information can be downloaded at https://www.mdpi.com/article/10.3390/molecules30071632/s1, Tables S1 and S2: Details of ECD calculations of (±)-**1**; Figure S1: Key molecular orbitals of (±)-**1**; Figures S2 and S3: Chiral separations of (±)-**1**; Table S3: NMR data for **2**, **4**, and **5**; Figures S4–S32: UV, IR, HRESIMS, 1D NMR, and 2D NMR spectra of compounds and synthetic products.
