# Supplementary material for: Polyketides with Cardioprotective Bioactivities from Sponge-Associated Fungus Aspergillus giganteus MA46-5
Source: Molecules. 2025 Apr 6;30(7):1632. doi: 10.3390/molecules30071632 (PMC11990167; doi:10.3390/molecules30071632)
Supplement: Supplementary file 1 [file molecules-30-01632-s001.zip › Supporting Information.pdf]

Supporting Information

Polyketides with cardioprotective bioactivities from sponge-associated fungus *Aspergillus giganteus* MA46-5

Ying-Tong Lin†, Xiao-Wei Yao†, Zheng-Wu Luo, Wei-Xin Jiang, Yin-Fei Wu, Ze-Jun Li, Xue-Wei Duan, Meng-Dan Zhang, Yuan-Yuan Cheng* and Cui-Xian Zhang*

School of Pharmaceutical Sciences, Guangzhou University of Chinese Medicine, Guangzhou 510006, P. R. China;

*Correspondence: zhangcuixian@gzucm.edu.cn (C.-X. Z.); chengyuanyuan@gzucm.edu.cn (Y.-Y. C.);

Tel.: +86-20-39358920 (C.-X.Z.); +86-20-39358684 (Y.-Y. C.)

†These authors contributed equally to this work.

Contents

Experimental Section

Quantum chemical ECD calculation of (±)-**1** S1-S2

Chiral separations of (±)-**1** S3

Spectral Information

Table S1 Cartesian coordinate of dominant conformer of (±)-**1**........ S1

Table S2 Key transitions and their related rotatory and oscillator strengths of dominant conformer of (±)-**1**........ S1-S2

Table S3 NMR data for **2**, **4** and **5** S3

Figure S1 Key molecular orbitals involved in important transitions regarding the ECD spectrum of dominant conformer of (±)-**1**........ S2

Figure S2 Chiral HPLC Chromatogram of natural product (±)-**1**........ S3

Figure S3 Chiral HPLC Chromatogram of synthetic product (±)-**1**........ S3

Figure S4. UV spectrum of **1** in MeOH. S4

Figure S5. IR spectrum of **1** S4

Figure S6. HRESIMS spectrum of **1** S4

Figure S7-S13. NMR data for **1** in $CDCl_3$ and DMSO-d_6........ S5-S8

Figure S14. IR spectrum of **3**........ S8

Figure S15. HRESIMS spectrum of **3**........ S8

Figure S16-S22. NMR data for **3** in DMSO-d_6 and $CDCl_3$........ S9-S12

Figure S23-S25. ^{1}H NMR data for **2**, **4** and **5**........ S12-S13

Figure S26. HRESIMS spectrum of synthetic product **1**........ S13

Figure S27-S28. NMR data for synthetic product **1** in $CDCl_3$........ S14

Figure S29-S30. NMR data for synthetic product **2** in Methanol-d_4........ S15

Figure S31-S32. NMR data for synthetic product **6** in $CDCl_3$........ S16

Experimental Section

1.Quantum chemical ECD calculation of (±)-1.

Table S1. Cartesian coordinate of dominant conformer of (±)-**1** (B3LYP/6-31+g(d)).

Standard orientation					
Center Number	Atomic Number	Atomic Type	Coordinates (Angstroms)		
			X	Y	Z
1	6	0	-3.222274	-0.843556	0.052929
2	6	0	-3.015578	0.538752	0.02846
3	6	0	-1.716683	1.040509	-0.01131
4	6	0	-0.610696	0.181998	-0.02914
5	6	0	-0.830943	-1.204066	-0.022222
6	6	0	-2.128729	-1.710068	0.024595
7	8	0	0.646511	0.715665	-0.068663
8	6	0	1.689866	-0.210622	0.258536
9	8	0	1.546018	-1.4038	-0.465614
10	6	0	0.374916	-2.117046	-0.079305
11	6	0	3.009763	0.443177	-0.100005
12	6	0	4.229336	-0.383776	0.324172
13	6	0	5.552605	0.298612	-0.036243
14	17	0	-1.467147	2.773735	-0.034507
15	8	0	-4.472306	-1.4011	0.096432
16	1	0	-3.853236	1.230243	0.043123
17	1	0	-2.307678	-2.781603	0.026932
18	1	0	1.61532	-0.422798	1.339754
19	1	0	0.537587	-2.611965	0.89272
20	1	0	0.237175	-2.899195	-0.831991
21	1	0	3.013256	0.607139	-1.184516
22	1	0	3.029591	1.431108	0.375843
23	1	0	4.192499	-0.559558	1.408868
24	1	0	4.176014	-1.369946	-0.151361
25	1	0	6.409412	-0.309176	0.275179
26	1	0	5.633312	0.459284	-1.118109
27	1	0	5.642306	1.277102	0.451279
28	1	0	-5.13533	-0.693745	0.098863

Table S2. Key transitions and their related rotatory and oscillator strengths of dominant conformer of (±)-**1**.

**HOMO is 116**					
**No.**	Energy (cm^{-1})	Wavelength (nm)	R(length)	Osc. Strength	Major contribs
**1**	38027.42822	262.9680856	-0.1959	0.1079	HOMO->LUMO (89%)
**2**	45492.08946	219.818437	-0.4021	0.0298	H-1->LUMO (17%), HOMO->L+1 (47%), HOMO->L+3 (16%)
**3**	46293.80456	216.0116261	7.1369	0.009	H-1->LUMO (20%), HOMO->L+1 (31%), HOMO->L+3 (26%)
**4**	47179.40132	211.9569074	-0.0239	0.0008	HOMO->L+2 (63%)
**5**	50541.12018	197.8586933	-13.8517	0.5062	H-1->LUMO (43%), HOMO->L+2 (14%), HOMO->L+3 (17%)
**6**	51618.6769	193.7283286	52.2058	0.2495	H-1->LUMO (14%), HOMO->L+3 (10%), HOMO->L+7 (17%), HOMO->L+8 (21%)
**7**	53034.17992	188.5576437	-2.8969	0.0326	H-1->L+1 (40%), H-1->L+2 (13%)

**8**	53849.60645	185.7023785	-64.7366	0.1287	H-1->L+3 (26%), HOMO->L+4 (14%), HOMO->L+6 (19%)
**9**	54021.40255	185.1118173	25.5294	0.1408	H-1->L+3 (18%), HOMO->L+4 (31%), HOMO->L+7 (16%)
**10**	54370.64061	183.9227916	22.4549	0.1437	H-1->L+3 (18%), HOMO->L+6 (39%)
**11**	55359.47634	180.6375468	38.3017	0.0525	H-1->L+1 (33%), H-1->L+2 (31%)
**12**	56922.57883	175.6772129	-2.0742	0.0074	H-2->LUMO (27%), OMO->L+7 (12%), HOMO->L+8 (21%), OMO->L+9 (13%)
**13**	56977.42453	175.5081084	-2.5459	0.0317	H-2->LUMO (56%), HOMO->L+9 (13%)
**14**	57722.68082	173.242127	-11.3776	0.0091	HOMO->L+4 (18%), HOMO->L+7 (15%), HOMO->L+9 (31%)
**15**	59179.31812	168.9779524	-1.9701	0.0009	HOMO->L+5 (25%), HOMO->L+6 (13%), HOMO->L+9 (15%), HOMO->L+10 (13%), HOMO->L+11 (10%)
**16**	60235.90442	166.0139429	-5.292	0.0038	H-1->L+4 (35%)
**17**	60256.87484	165.9561673	2.753	0.0121	HOMO->L+10 (15%), HOMO->L+11 (23%), HOMO->L+16 (12%)
**18**	60678.7028	164.8024684	-2.5741	0.002	H-1->L+2 (31%), H-1->L+7 (29%), H-1->L+8 (10%)
**19**	60997.2918	163.9417047	14.1678	0.0178	H-3->LUMO (60%)
**20**	61933.70149	161.4629799	5.3061	0.0066	H-4->LUMO (35%), H-2->L+3 (17%)
**21**	62181.3137	160.8200182	9.1891	0.0037	H-4->LUMO (27%), H-2->L+3 (19%)
**22**	62312.78208	160.4807179	-4.3518	0.0086	H-4->LUMO (21%), H-1->L+6 (18%), HOMO->L+13 (27%)
**23**	63085.46122	158.5151286	6.9069	0.0022	HOMO->L+5 (19%), HOMO->L+10 (13%)
**24**	63211.28371	158.1996032	-2.584	0.0029	HOMO->L+5 (11%), HOMO->L+11 (12%), HOMO->L+13 (20%)
**25**	63396.79123	157.7366899	8.7634	0.0109	H-4->L+1 (18%), H-4->L+2 (11%), H-4->L+8 (11%)
**26**	63684.73116	157.0235097	10.4509	0.0315	H-1->L+9 (22%), HOMO->L+13 (14%)
**27**	64136.40164	155.9176964	3.5169	0.0009	H-1->L+9 (20%), HOMO->L+12 (25%)
**28**	64329.9747	155.44853	-9.5809	0.0126	H-2->L+3 (11%), H-2->L+4 (32%), H-1->L+8 (10%)
**29**	64833.26467	154.2418086	-1.4062	0.0381	H-5->LUMO (58%)
**30**	65316.39077	153.1009274	-5.0095	0.0063	HOMO->L+14 (23%)

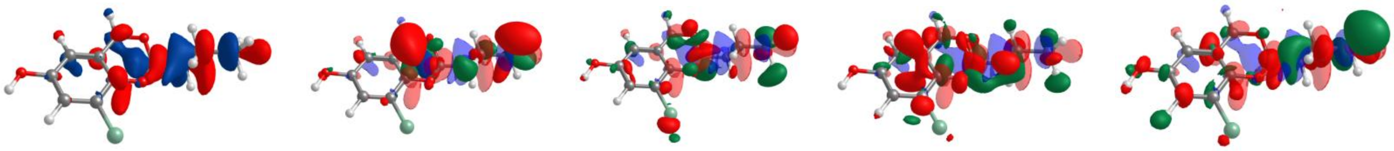

115 **116（HOMO）** **117(LUMO）** **118** **120**

Figure S1. Key molecular orbitals involved in important transitions regarding the ECD spectrum of dominant conformer of (±)-**1**.

2.**Chiral separations of (±)-1.**

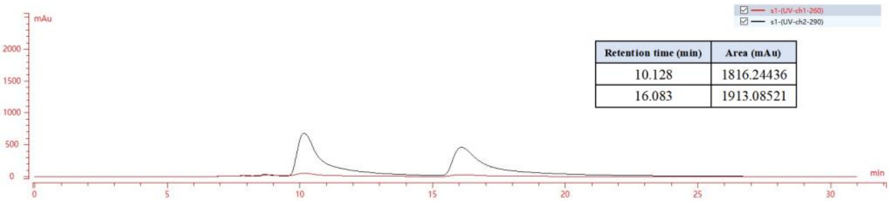

Figure S2. Chiral HPLC Chromatogram of natural product (±)-**1** (Chiral ND (2) NFC 5u, isopropanol–n-hexane = 30/70, flow rate = 2.0 mL/min).

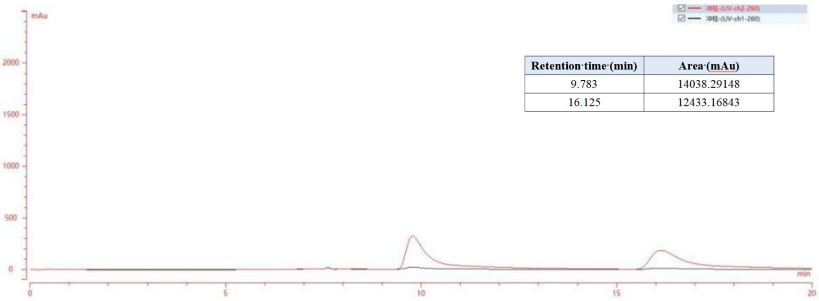

Figure S3. Chiral HPLC Chromatogram of synthetic product (±)-**1** (Chiral ND (2) NFC 5u, isopropanol–n-hexane = 30/70, flow rate = 2.0 mL/min).

Spectral Information

Table S3. ^{1}H and ^{13}C NMR spectral data of **2** in DMSO-d_6, **4** in $CDCl_3$ and **5** in DMSO-d_6 (δ in ppm, J in Hz).

No.	**2**		**4**		**5**	
	δ_C, type	δ_H, mult, J	δ_C, type	δ_H, mult, J	δ_C, type	δ_H, mult, J
1	132.8, C		110.4, C			
1a					156.2, C	
2	141.8, C		158.9, C		138.3, C	
3	120.6, C		113.5, C		86.7, CH	5.58, s
4	113.5, CH	6.60, d, (2.9)	161.4, C		169.4, C	
4a					106.1, C	
5	150.6, C		114.6, C		161.8, C	
6	113.0, CH	6.73, d, (2.9)	130.0, CH	7.37, s	100.5, CH	6.54, d, (2.3)
7	58.8, CH_2	4.46, s	202.9, C		160.4, C	
8			26.4, CH_3	2.55, s	116.0, CH	6.57, d, (2.3)
9			7.6, CH_3	2.13, s	23.1, CH_3	2.53, s
10			15.7, CH_3	2.21, s	56.6, CH_3	3.92, s
2-OH		9.10, s		12.88, s		
4-OH				5.32, s		
5-OH		8.39, s				
7-OH		5.18, s				10.45, s

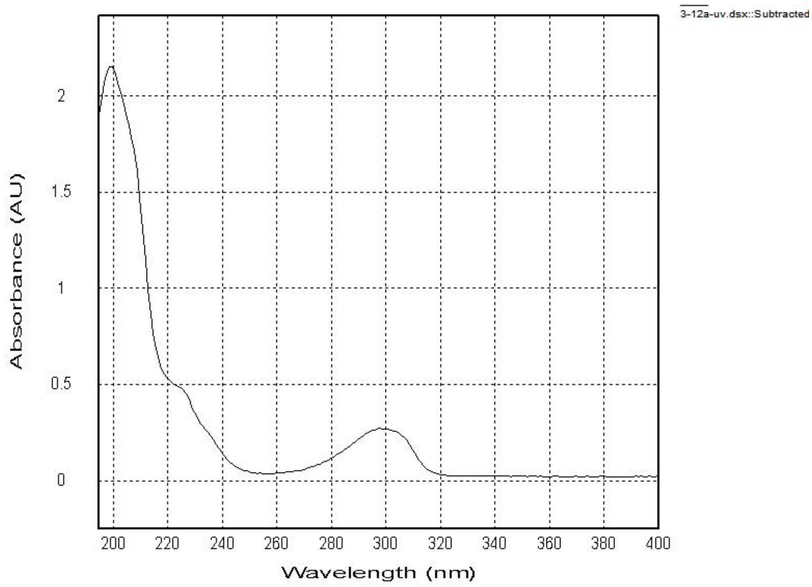

Figure S4. UV spectrum of **1** in MeOH.

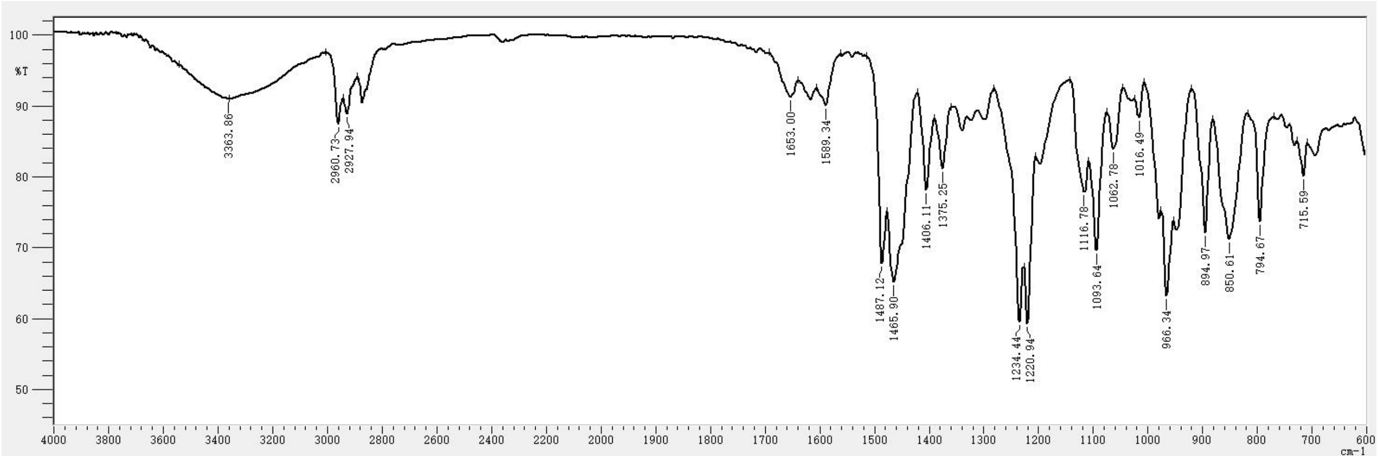

Figure S5. IR spectrum of **1**.

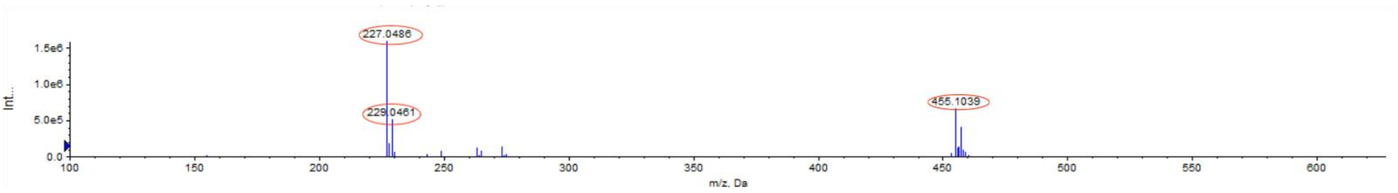

Figure S6. HRESIMS spectrum of **1**.

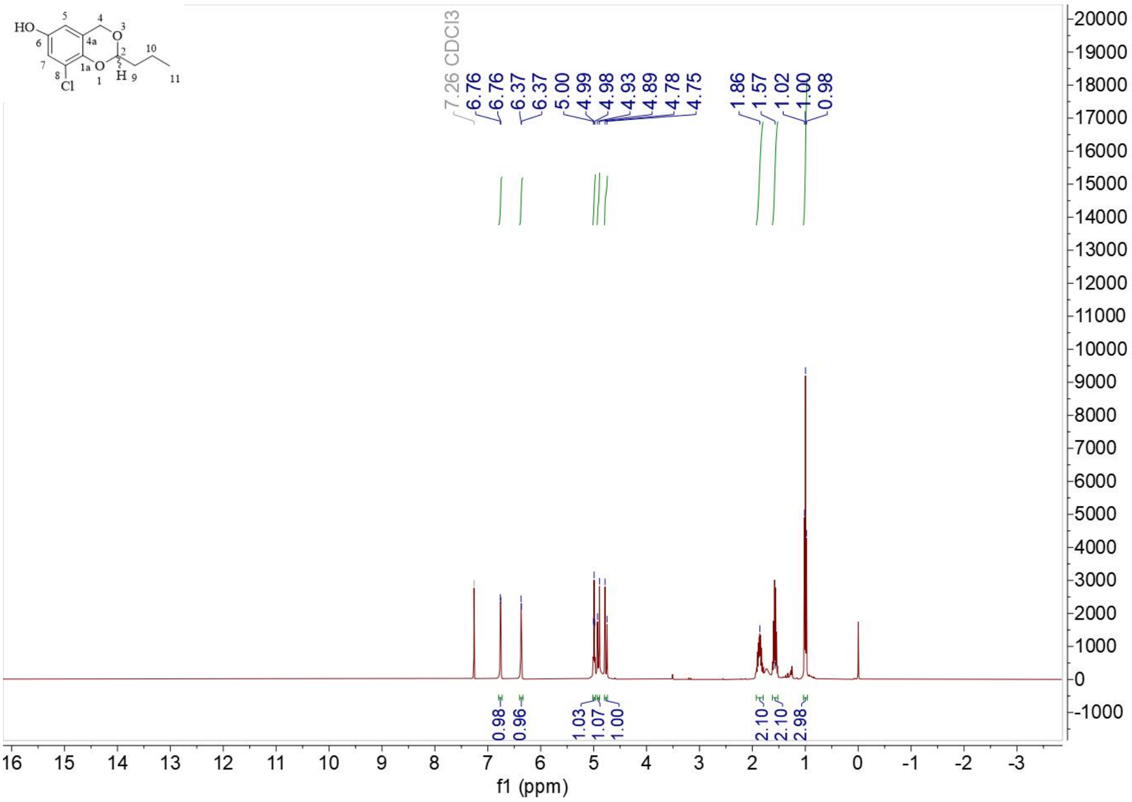

Figure S7. ^{1}H NMR spectrum of **1** in $CDCl_3$ (400 MHz).

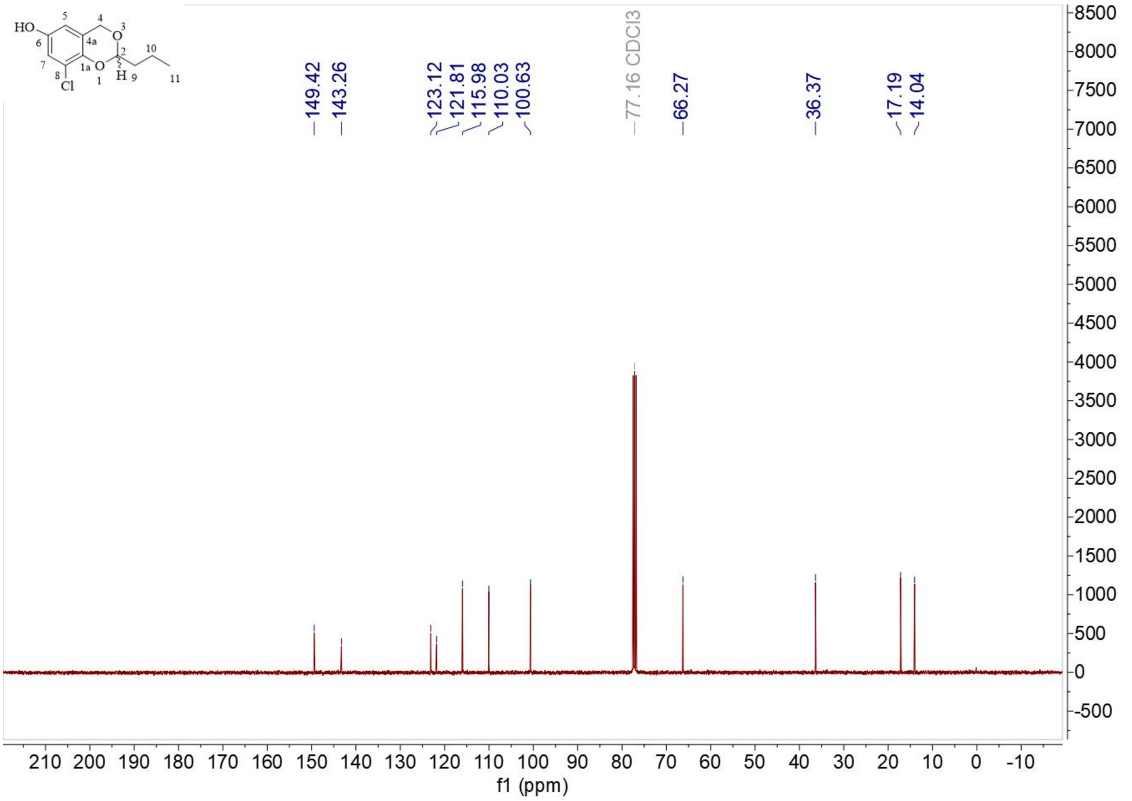

Figure S8. ^{13}C NMR spectrum of **1** in $CDCl_3$ (100 MHz).

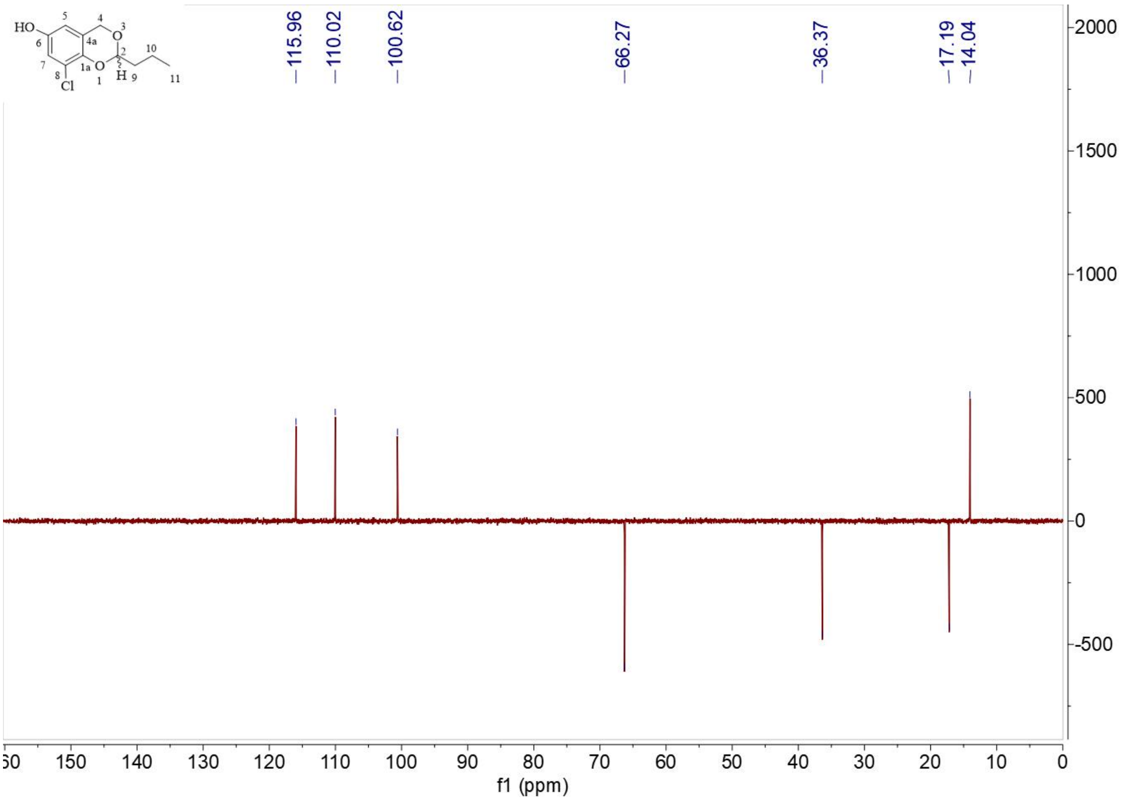

Figure S9. DEPT135 NMR spectrum of **1** in $CDCl_3$ (100 MHz).

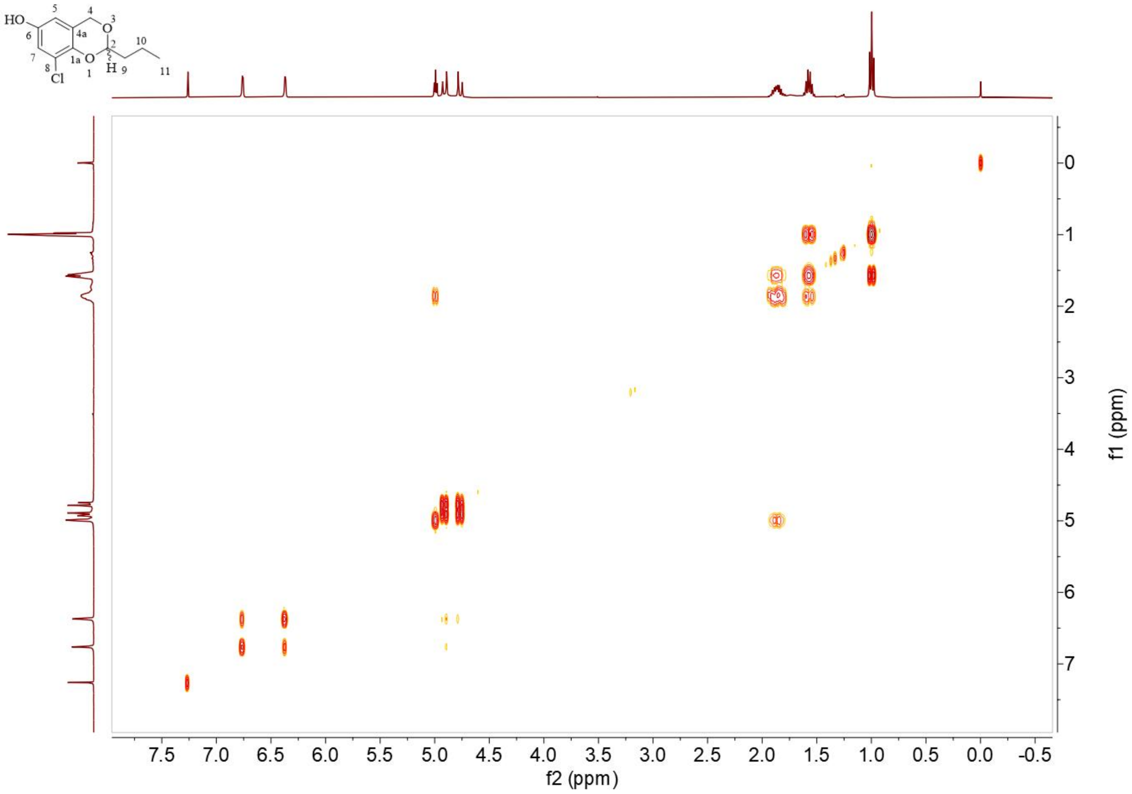

Figure S10. $^1H-^1H$ COSY spectrum of **1** in $CDCl_3$ (400 MHz).

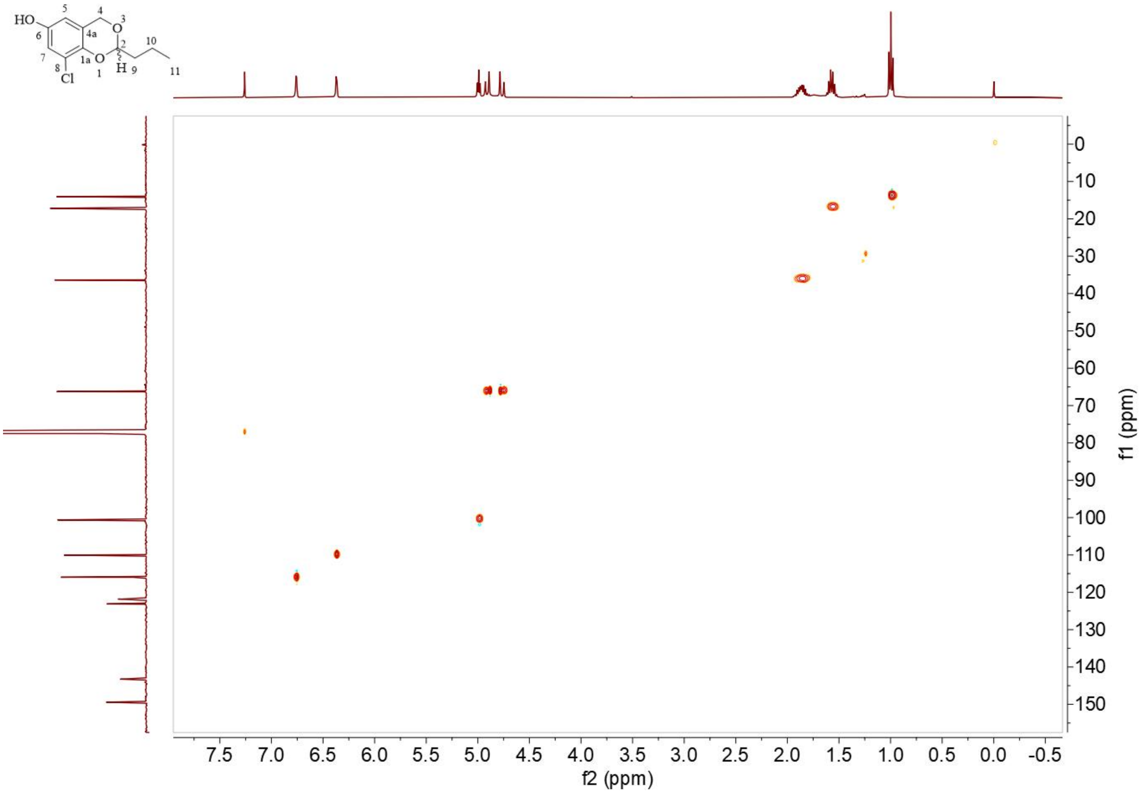

Figure S11. HSQC spectrum of **1** in $CDCl_3$ (400 MHz).

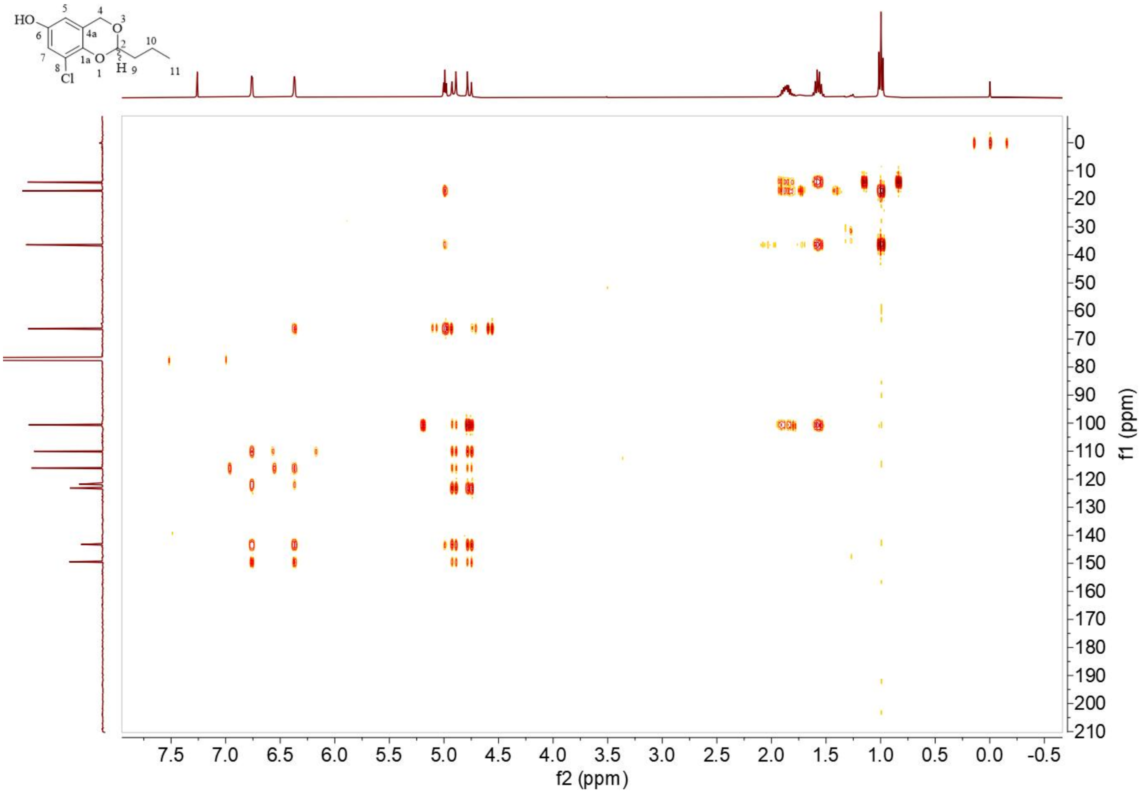

Figure S12. HMBC spectrum of **1** in $CDCl_3$ (400 MHz).

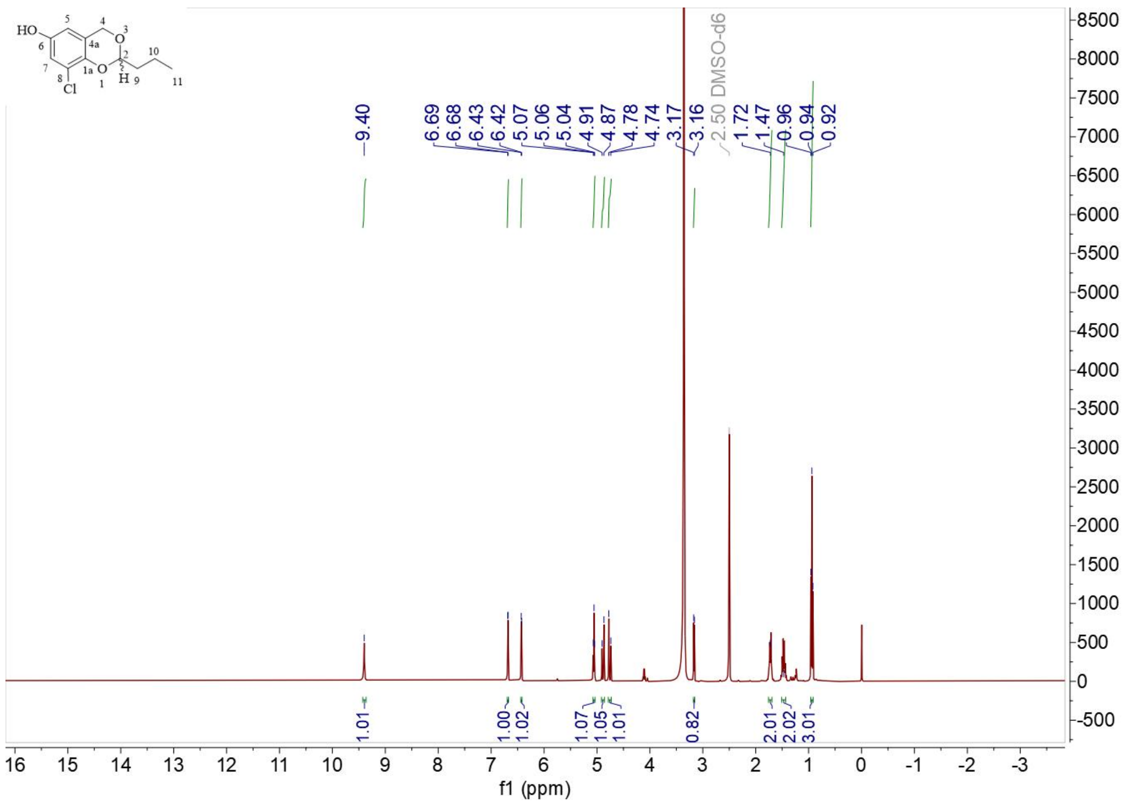

Figure S13. ^{1}H NMR spectrum of **1** in DMSO-d_6 (400 MHz).

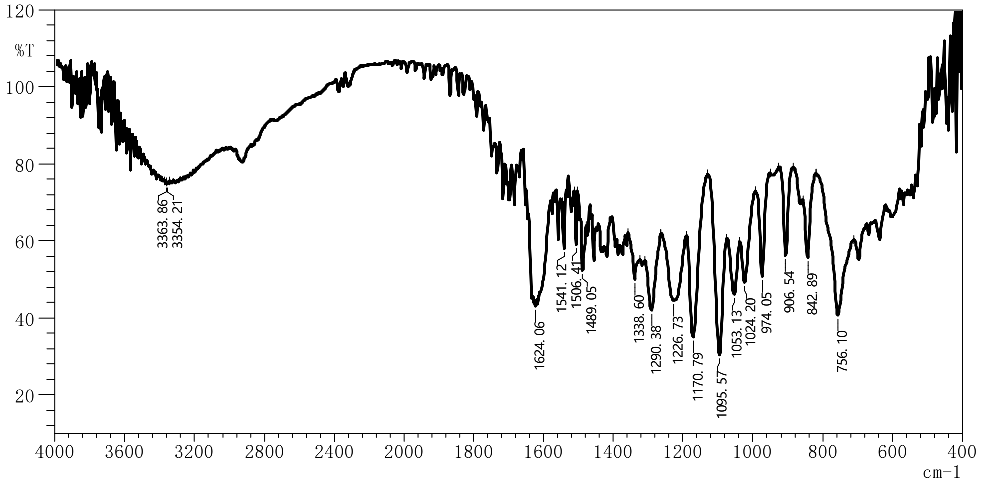

Figure S14. IR spectrum of **3** .

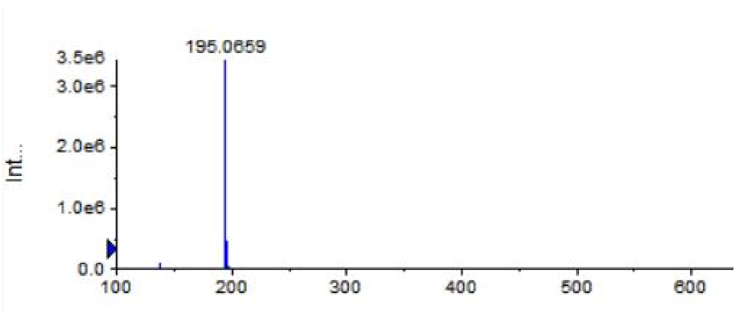

Figure S15. HRESIMS spectrum of **3**.

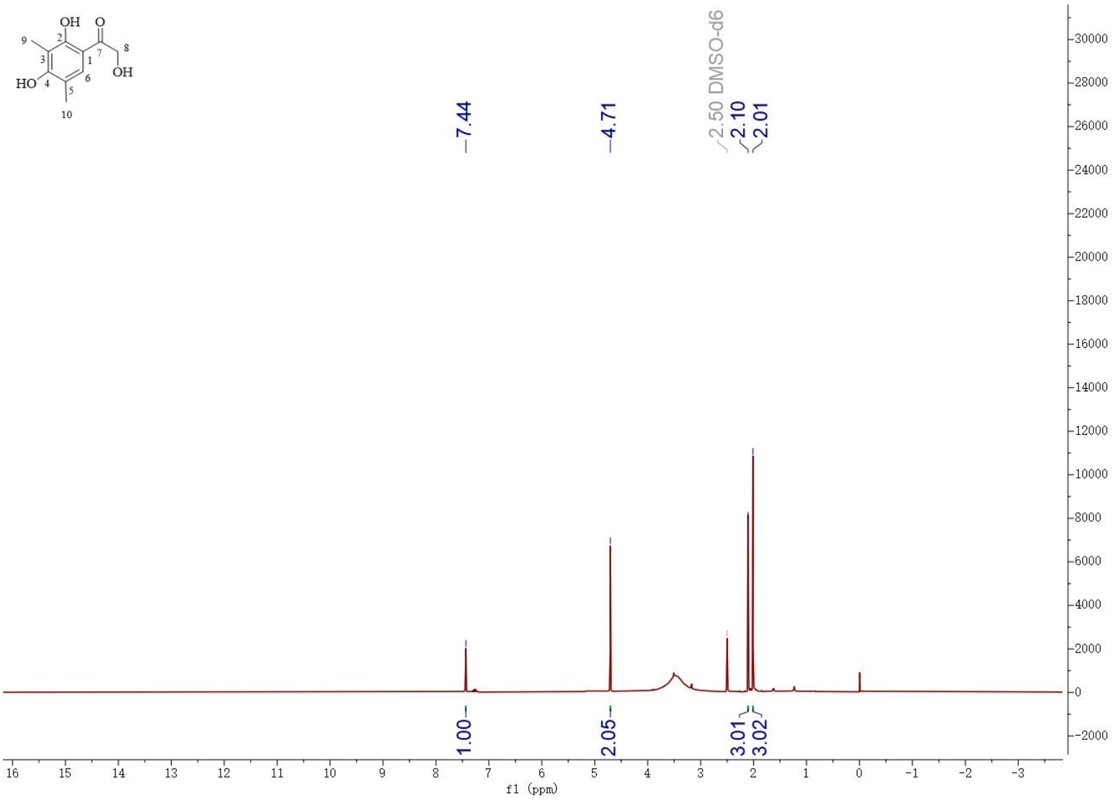

Figure S16. ^{1}H NMR spectrum of **3** in DMSO-d_6 (400 MHz).

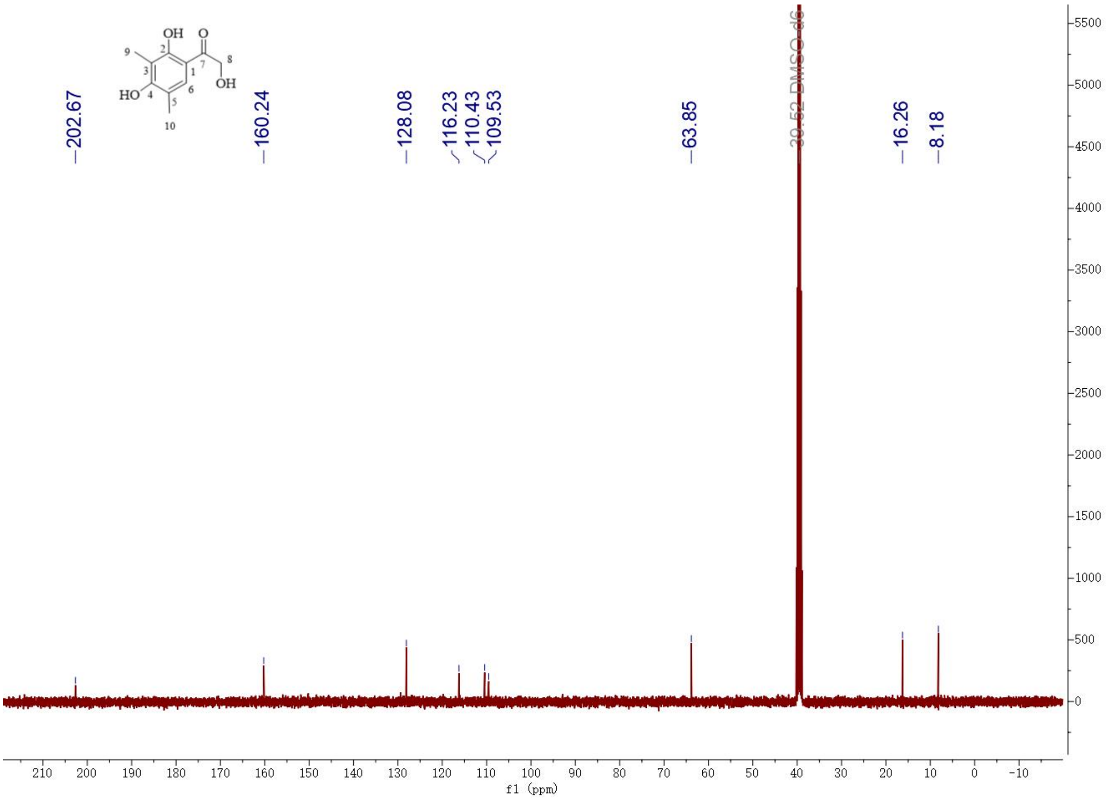

Figure S17. ^{13}C NMR spectrum of **3** in DMSO-d_6 (100 MHz).

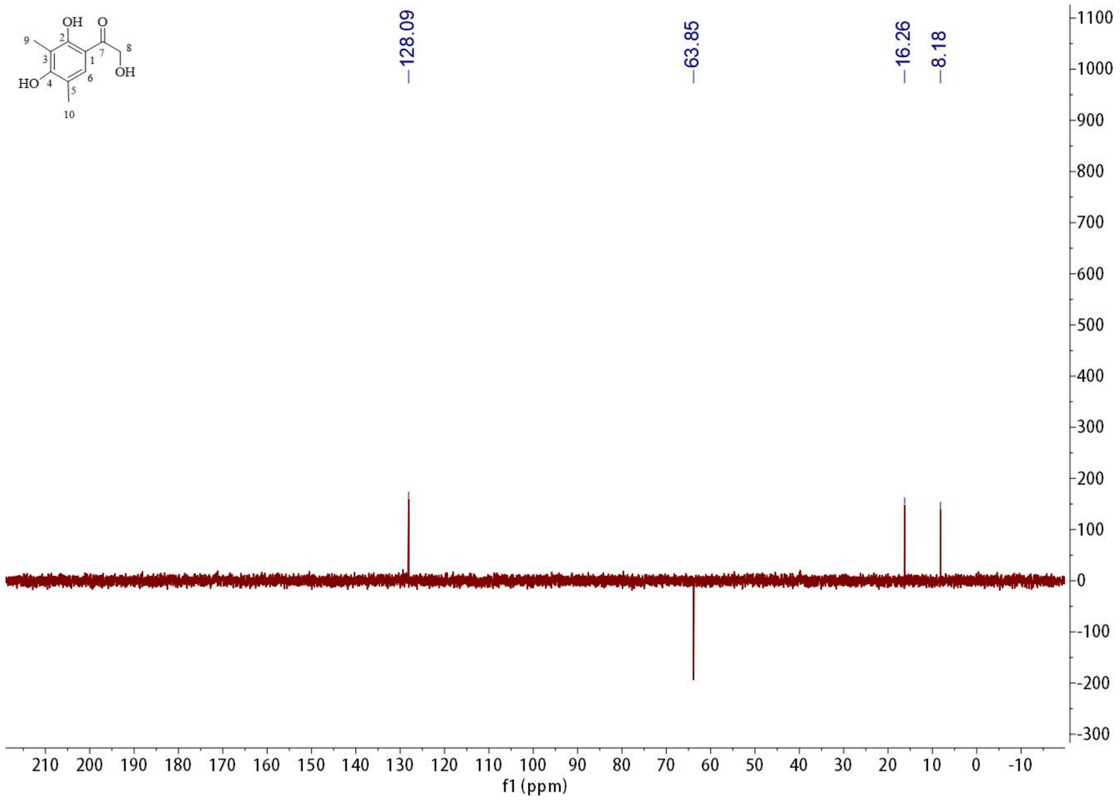

Figure S18. DEPT135 NMR spectrum of **3** in DMSO-d_6 (100 MHz).

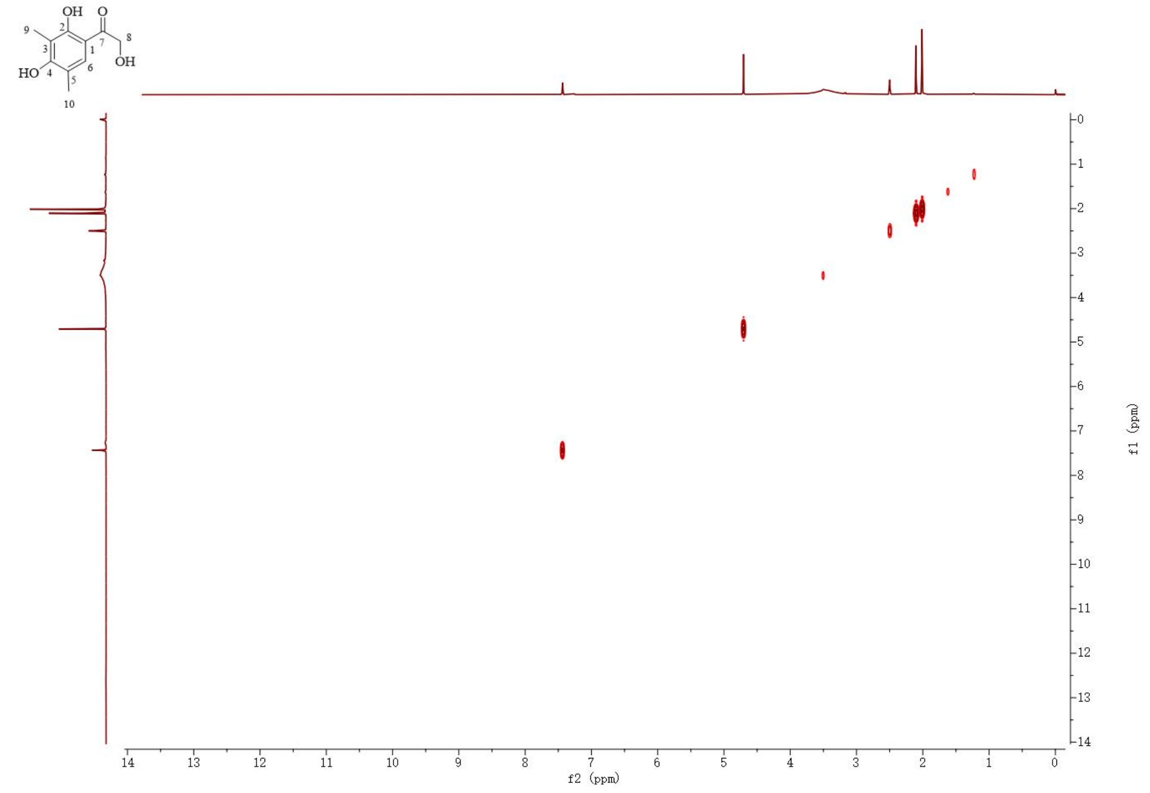

Figure S19. $^1H-^1H$ COSY spectrum of **3** in DMSO-d_6 (400 MHz).

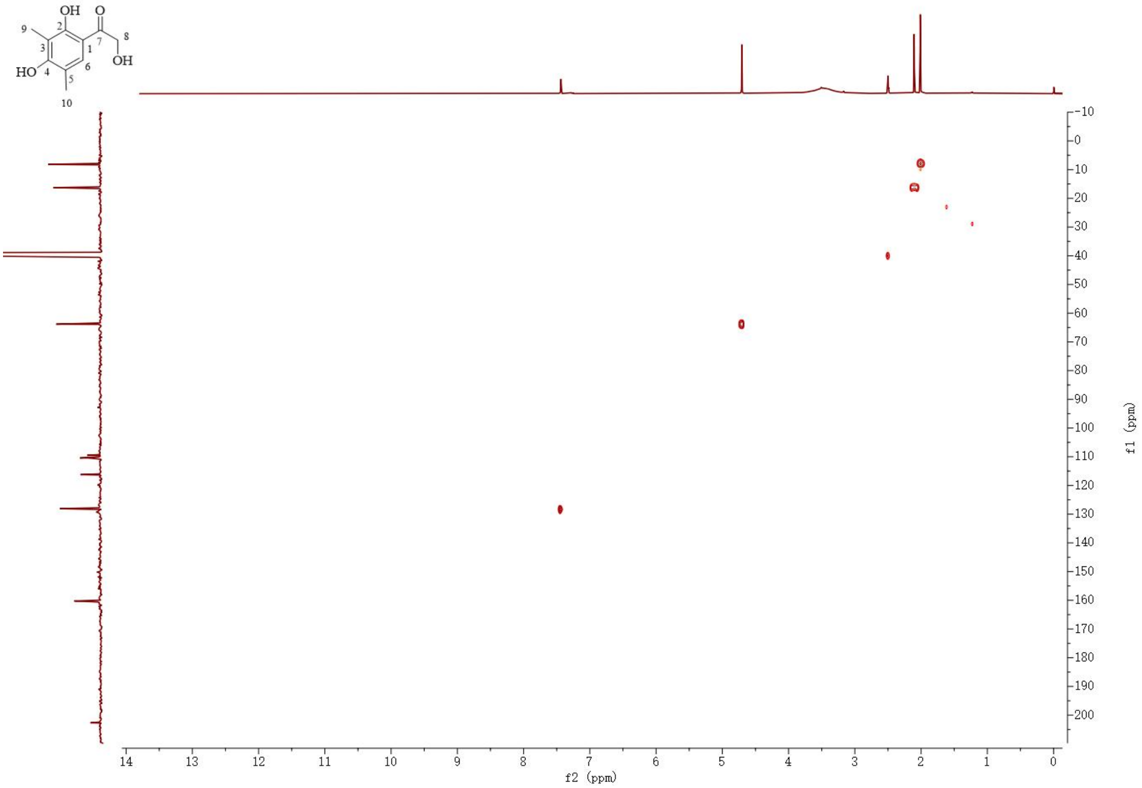

Figure S20. HSQC spectrum of **3** in DMSO-d_6 (400 MHz).

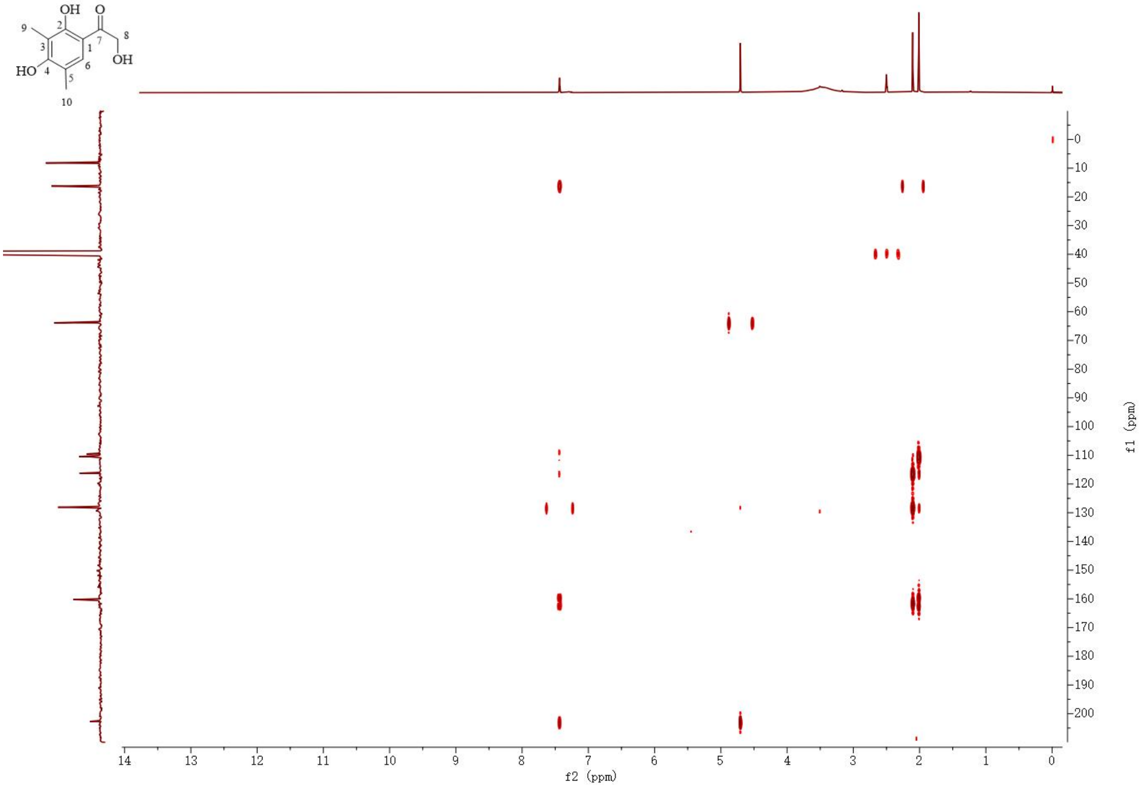

Figure S21. HMBC spectrum of **3** in DMSO-d_6 (400 MHz).

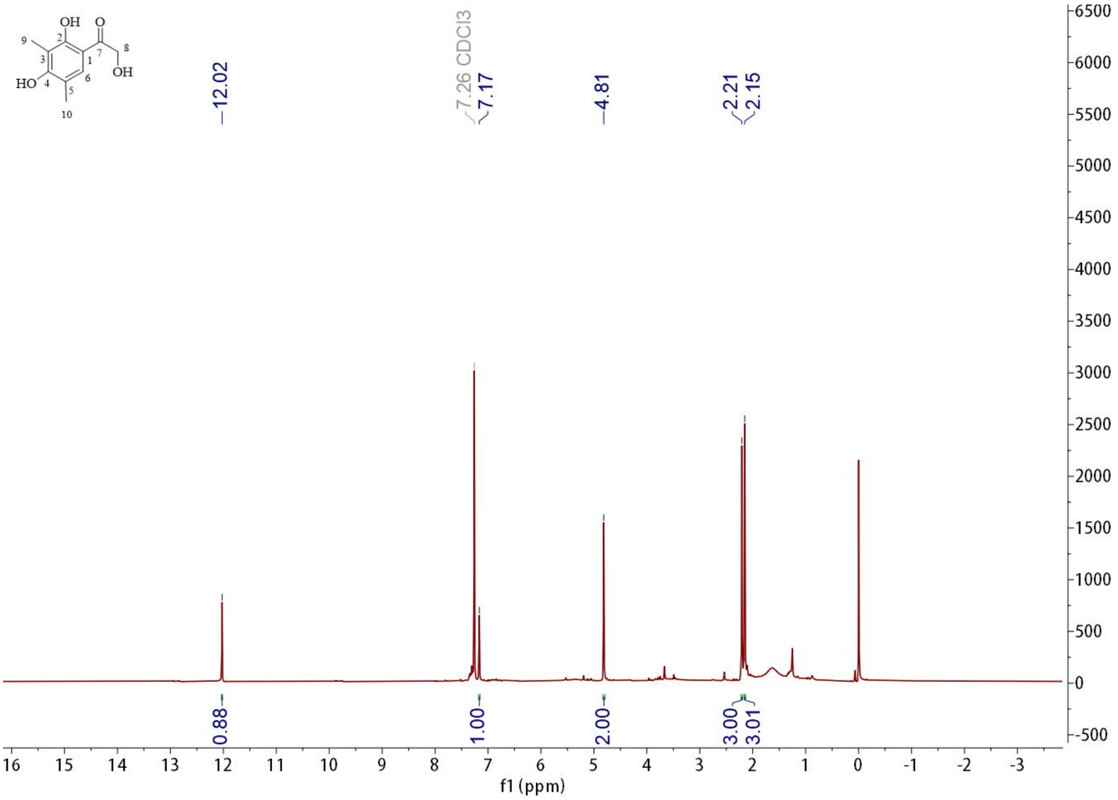

Figure S22. ^{1}H NMR spectrum of **3** in $CDCl_3$ (400 MHz).

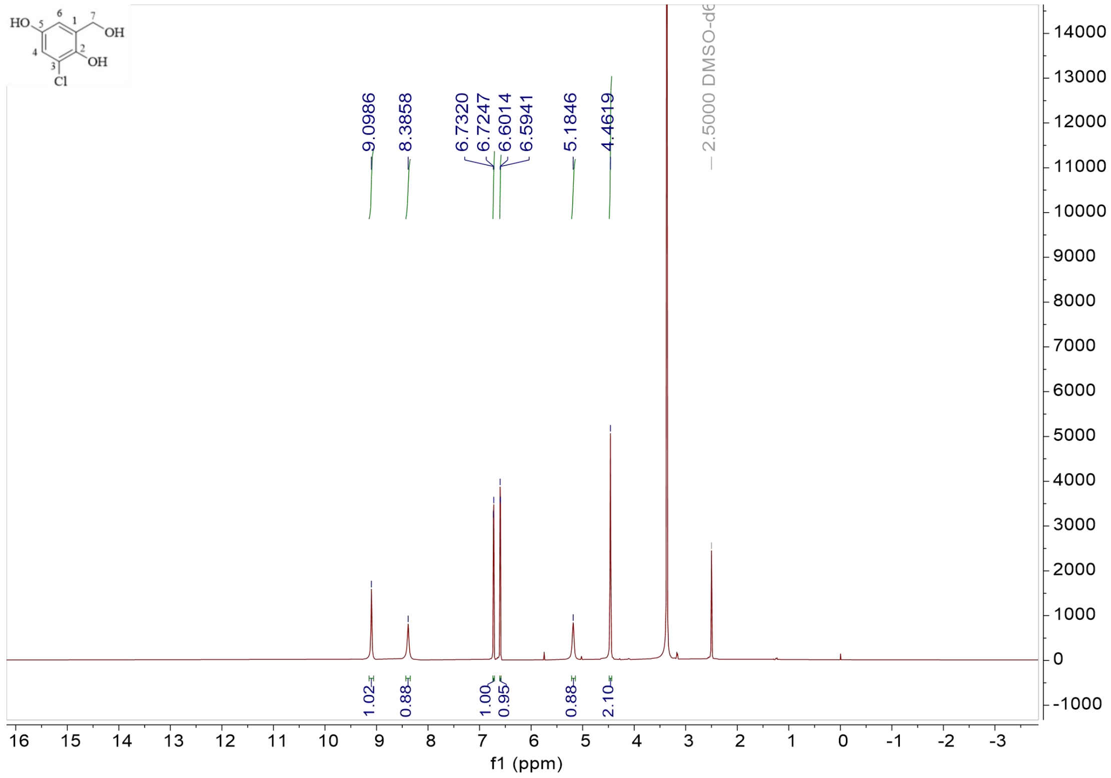

Figure S23. ^{1}H NMR spectrum of **2** in DMSO-d_6 (400 MHz).

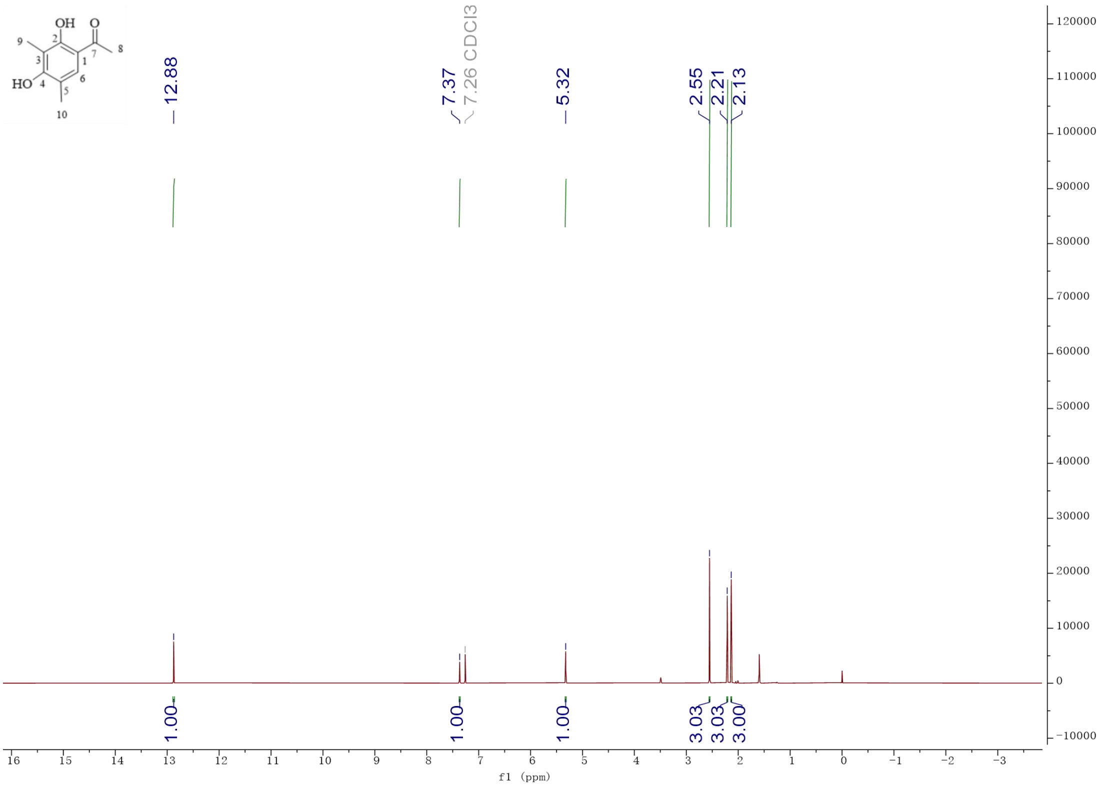

Figure S24. ^{1}H NMR spectrum of **4** in $CDCl_3$ (400 MHz).

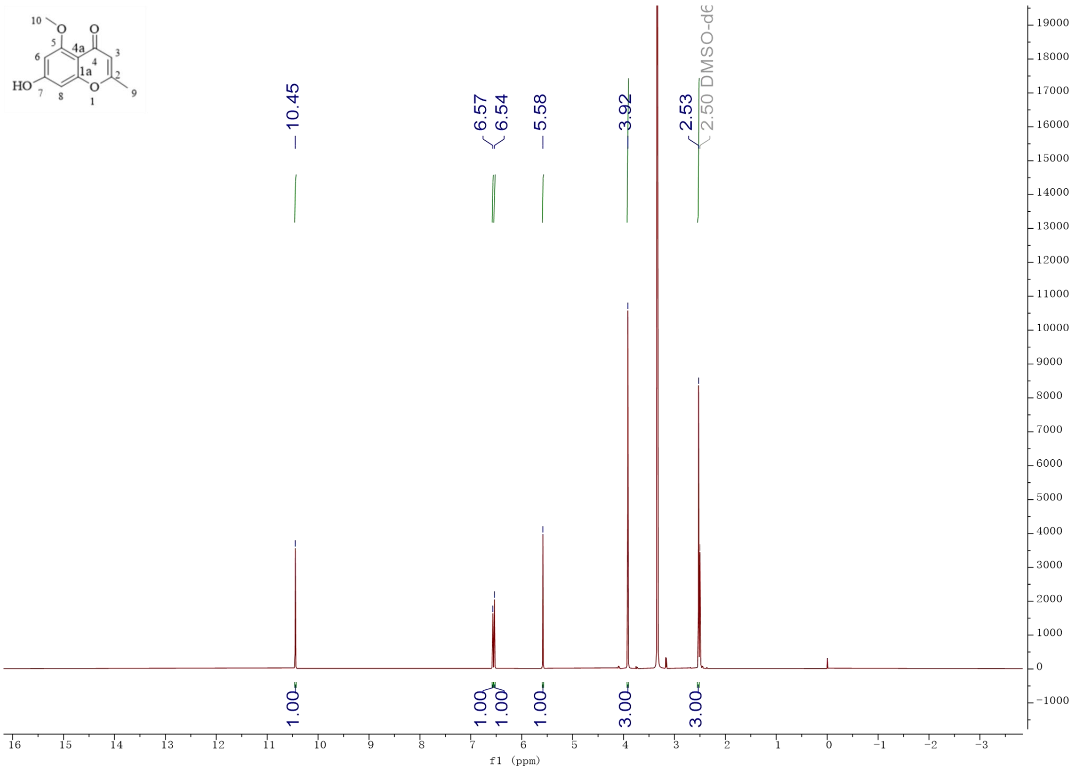

Figure S25. ^{1}H NMR spectrum of **5** in DMSO-d_6 (400 MHz).

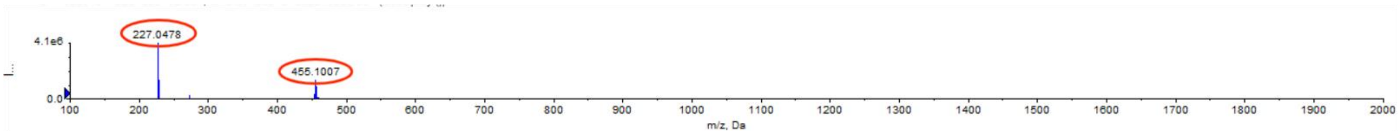

Figure S26. HRESIMS spectrum of synthetic product **1**.

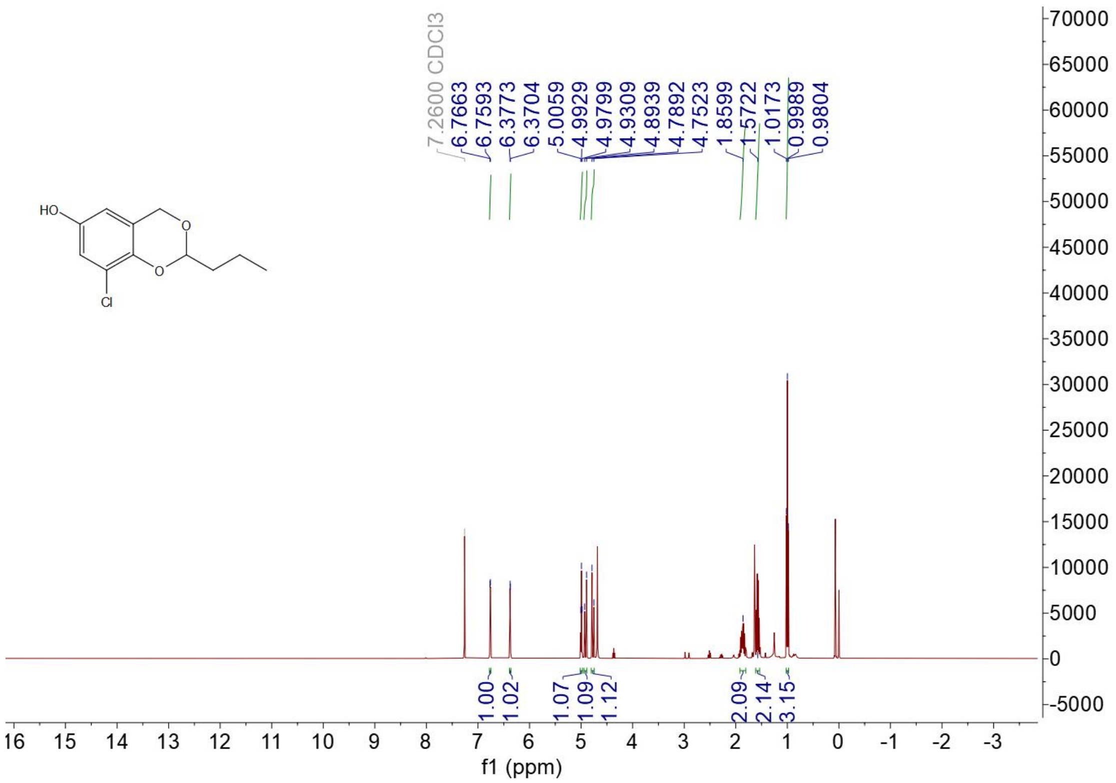

Figure S27. ^{1}H NMR spectrum of synthetic product **1** in $CDCl_3$ (400 MHz).

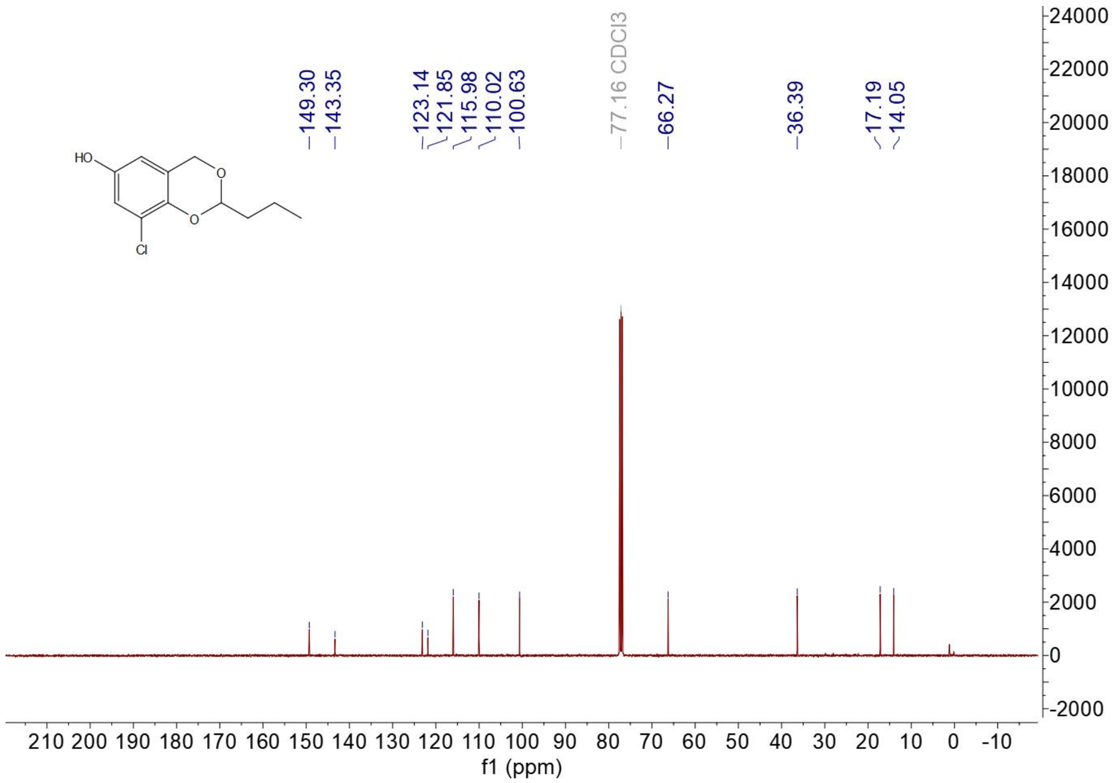

Figure S28. ^{13}C NMR spectrum of synthetic product **1** in $CDCl_3$ (100 MHz).

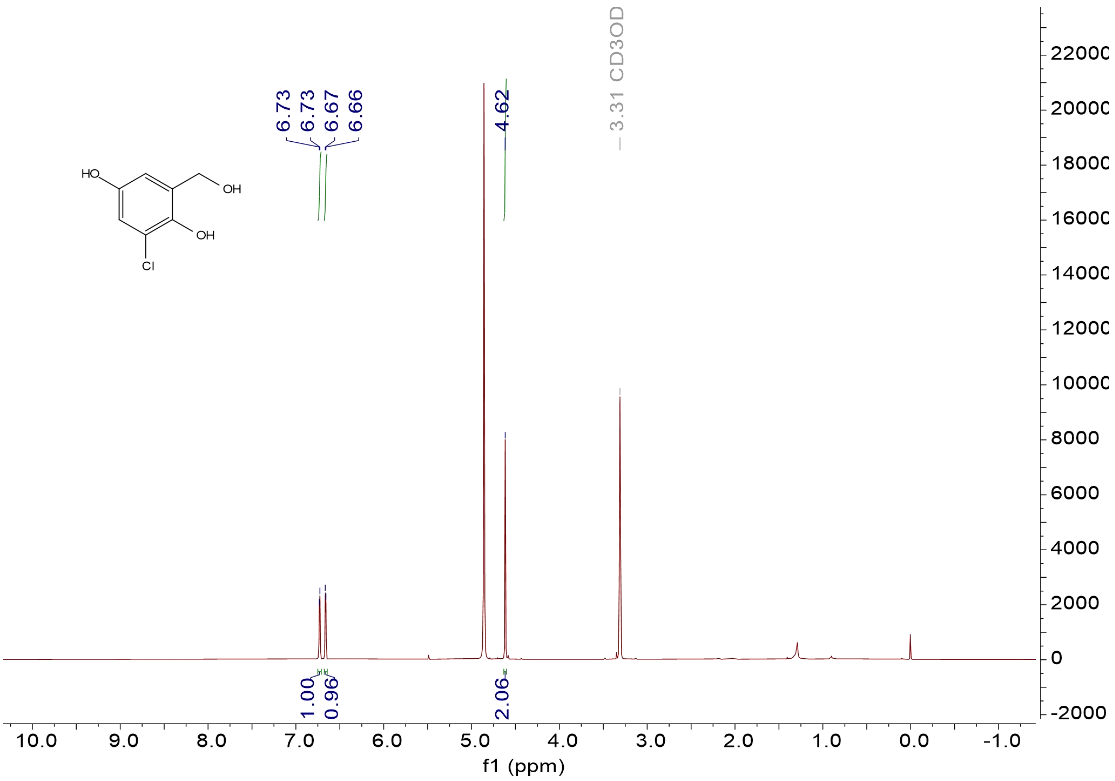

Figure S29. ^{1}H NMR spectrum of synthetic product **2** in Methanol-d_4 (400 MHz).

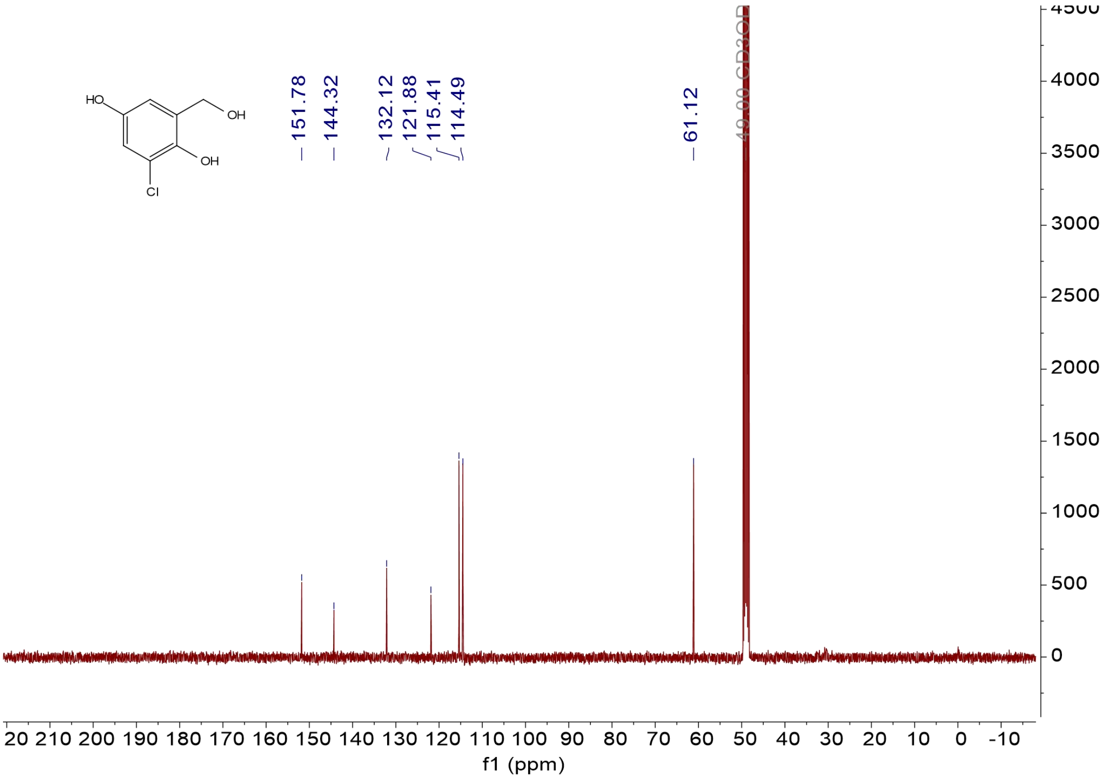

Figure S30. ^{13}C NMR spectrum of synthetic product **2** in Methanol-d_4 (100 MHz).

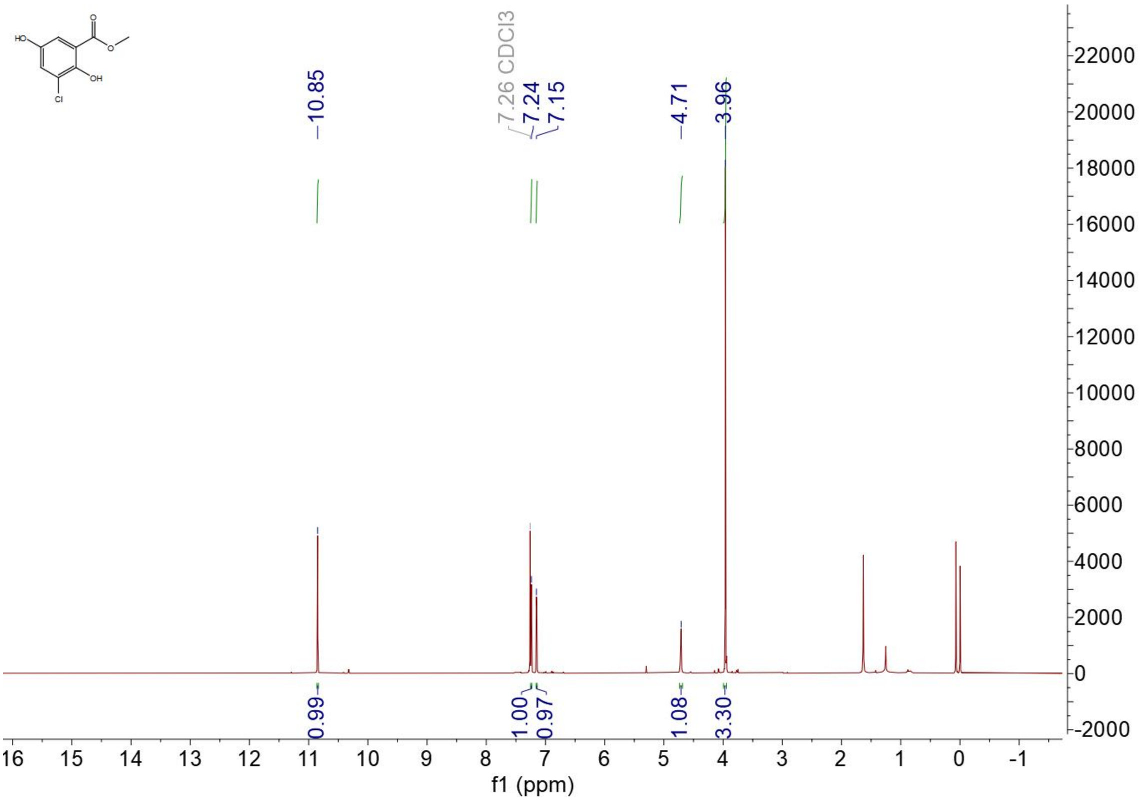

Figure S31. ^{1}H NMR spectrum of synthetic product **6** in $CDCl_3$ (400 MHz).

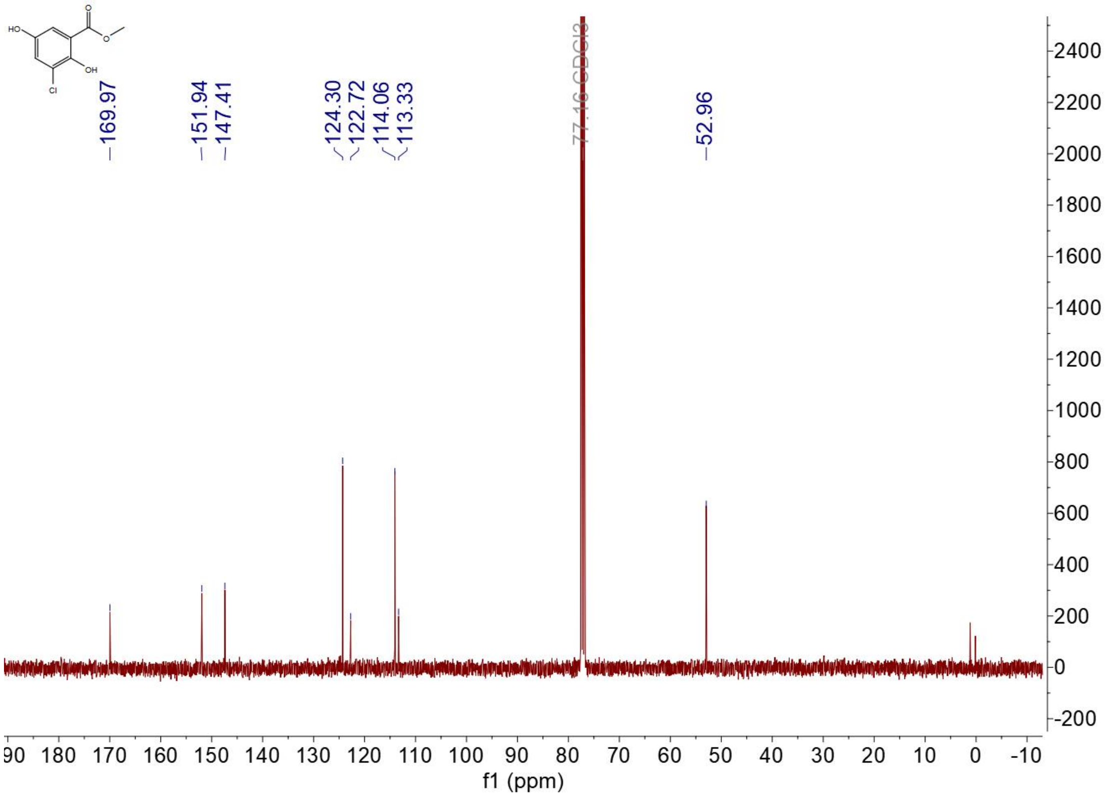

Figure S32. ^{13}C NMR spectrum of synthetic product **6** in $CDCl_3$ (100 MHz).
